# Clinician and patient views on janus kinase inhibitors in the treatment of inflammatory arthritis: a mixed methods study

**DOI:** 10.1186/s41927-023-00370-7

**Published:** 2024-01-17

**Authors:** Savia de Souza, Ruth Williams, Elena Nikiphorou

**Affiliations:** 1 Centre for Rheumatic Diseases, King’s College London, Weston Education Centre, London, SE5 9RJ UK; 2 Rheumatology Department, King’s College Hospital, London, SE5 9RS UK

**Keywords:** JAK inhibitors, Clinical practice, Patient perspective, Inflammatory arthritis, Rheumatoid arthritis, Psoriatic arthritis, Surveys, Focus groups, Mixed methods, COVID-19

## Abstract

**Background:**

Janus kinase inhibitors (JAKi) are new targeted synthetic disease-modifying antirheumatic drugs (DMARDs) licenced in the UK to treat rheumatoid and psoriatic arthritides. Unlike currently often prescribed biological DMARDs, they target a different part of the inflammatory pathway and are taken orally. The aim of this study was to explore what UK-based rheumatology clinicians and inflammatory arthritis (IA) patients think about the awareness, prescription and use of JAKi; how they compare with biologics; and how the COVID-19 pandemic has affected how JAKi are viewed and prescribed.

**Methods:**

Rheumatology clinicians and IA patients completed online surveys and participated in interviews/focus groups between September 2021 and January 2022. Survey data were analysed descriptively, and interview/focus group data underwent an inductive thematic analysis.

**Results:**

66.6% of patients had at least some awareness of JAKi, 73.0% from their rheumatology team. Problems getting earlier access to these drugs were raised by some patients, with many being prescribed JAKi after multiple other therapies had failed. 91.5% of clinicians prescribed JAKi in keeping with their local guidelines, with 72.3% prescribing them frequently as a monotherapy. Some clinicians had lingering safety concerns over JAKi use. Despite experiencing side effects and knowing of possible long-term risks, patients felt overall the benefits of JAKi outweighed the risks. 39.3% of patients were ‘very satisfied’ on JAKi, compared with 25.0% on biologics. Patients on JAKi appreciated their short half-life when it comes to infections, and their convenience as an oral therapy. When JAKi were discontinued in patients, it was predominantly due to inefficacy and non-cardiovascular adverse events. The COVID-19 pandemic resulted in increased prescription of JAKi as an alternative to injections and infusions, primarily to avoid potentially exposing patients to the coronavirus. Some patients believed their JAKi may confer some protection against developing severe COVID-19.

**Conclusion:**

JAKi are an effective treatment option for IA and are liked by patients. The COVID-19 pandemic appears to have impacted their prescription favourably. However, clinicians have safety concerns over JAKi use. Any decision to go on a JAKi should be informed and take into account individual patient risk factors, circumstances and preferences.

**Supplementary Information:**

The online version contains supplementary material available at 10.1186/s41927-023-00370-7.

## Background

Janus kinase inhibitors (JAKi) belong to one of the newest classes of rheumatology drugs for people with rheumatoid arthritis (RA) and psoriatic arthritis (PsA). Tofacitinib and baricitinib were the first JAKi to be licensed in the UK in 2017, followed by upadacitinib in 2019 and filgotinib in 2020 [1]. Tofacitinib and upadacitinib are licensed to treat moderate to severe RA and active PsA, whilst baricitinib and filgotinib are licensed for use in moderate to severe RA [2–5].

This newest class of targeted synthetic disease-modifying antirheumatic drugs (tsDMARDs) differ from often currently prescribed biological DMARDs (bDMARDs/biologics) in two key ways: i) they target a different part of the inflammatory pathway (the janus kinase-signal transducer and activator of transcription pathway), and ii) are taken orally (rather than as an intravenous infusion or subcutaneous injection) [6]. Consequently, they are especially useful for patients who have been unsuccessful on other therapies, experience injection site reactions, have poor manual dexterity or are needle-phobic [6–8].

Being taken orally, JAKi remove the need for patients to attend hospitals for infusions, injection training visits, and make life and travel generally easier. Thus, minimising individual burden. There is also a potential cost saving to be made to the UK National Health Service with a shift from biologic infusions (resource-intensive) and injections to oral JAKi, which will ultimately become cheaper than biosimilars once off patent (due to lower manufacturing costs) [9].

With a shorter half-life than biologics (including biosimilars), JAKi therapies can also be quickly stopped and restarted when patients have an infection or require surgery. Being able to rapidly reverse immunosuppression conferred advantages during the coronavirus disease 2019 (COVID-19) pandemic. Some JAKi have also been trialled as a treatment in severely ill COVID-19 patients [10].

However, the recent Oral Rheumatoid Arthritis Trial (ORAL) Surveillance study [11] has raised some important concerns over JAKi use. This study looked at moderate to severe RA patients aged 50 and over on tofacitinib who had at least one additional cardiovascular disease risk factor and no current or previous malignancy, and found them more likely to experience major adverse cardiovascular events (MACEs) and to develop cancer compared with patients on anti-tumour necrosis factor (anti-TNF) bDMARDs [11]. In this population, tofacitinib at a 10 mg twice daily dose was also found to be associated with a higher risk of death due to any cause, serious infections and venous thromboembolisms (VTEs) [11]. This led to the UK Medical Health and Regulatory Authority (MHRA) issuing a warning to limit the use of tofacitinib and, with uncertainty as to whether this was a class effect, the US Food and Drug Administration putting black box warnings on all licensed JAKi used for the treatment of inflammatory arthritis (IA) in the USA in late 2021 [12, 13]. In early 2022, The European Medicines Agency (EMA) started a safety review on all JAKi used in IA [14].

As newer drugs, JAKi do not have the evidence-base that biologics do, particularly from clinician and patient perspectives [15–17]. Although there are recent studies comparing the real-world effectiveness of JAKi with biologics, through registry data analysis of patients with RA [18, 19], there is currently a lack of published literature on what clinicians and IA patients think about the awareness, prescription and use of JAKi; how they compare with biologics; and, relevant to current times, how the COVID-19 pandemic has affected JAKi use and prescription. This study aimed to fill these knowledge gaps.

## Methods

A mixed methods approach was adopted consisting of surveys (which included free text responses), interviews and focus groups. All methods were carried out in accordance with relevant guidelines and regulations. Ethical approval for this study was granted by the UK National Health Service Health Research Authority Health and Social Care Research Ethics Committee A (reference number: 21/NI/0111).

### Surveys

Two surveys (one for clinicians and one for patients) were designed by the research team, which included two Patient Researchers with IA (SdS and RW). Both surveys provided information on the study at the start and survey completion implied consent to participate.

The clinician survey was piloted on four rheumatologists and one rheumatology nurse, from across the UK, and amended in accordance with their feedback. The final survey (see Additional file 1) was created online using SurveyMonkey, with the survey link advertised on Twitter in September 2021. Clinicians were asked to only complete the survey if they met the inclusion criteria: i) are a rheumatologist or rheumatology nurse specialist; ii) practice in the UK; and iii) regularly see patients with RA and/or PsA. The survey consisted of 15 items, with closed- and open-ended questions, and responses collected were anonymous.

The patient survey was piloted on two Patient Experts with IA (CS and TE) from the King’s College London Centre for Rheumatic Diseases and amended based on their feedback. Its main aim was to help recruit patients and establish further discussion areas for the pilot interviews and focus groups. In order to reduce patient burden/fatigue of filling in a lengthy survey, and therefore increase the completion rate, it was decided to keep it brief with the patient interviews/focus groups allowing for more in depth exploration of topics.

The final survey (see Additional file 2) was created online using SurveyMonkey, with the survey link advertised via Facebook, Twitter and UK-based patient charities (the National Rheumatoid Arthritis Society and the Psoriasis Association) in October 2021. Patients were asked to only complete the survey if they met the inclusion criteria: i) aged 18 + years, ii) live in the UK, iii) have a diagnosis of RA or PsA, and iv) are currently on a biologic/JAKi (≥ six months) or have previously been on JAKi therapy. The survey consisted of 23 items, with closed- and open-ended questions, and responses collected were anonymous. Patients were asked at the end of the survey whether they would be interested in participating in a focus group, provided they had current or previous experience of JAKi therapy.

### Interviews and focus groups

A topic guide for the patient interviews/focus groups (see Additional file 3) was devised by the research team and reviewed by the two departmental Patient Experts (CS and TE). The patient survey responses did not generate any further questions to be added to the topic guide. Eighteen patients were purposefully selected (to create as diverse a sample as possible), from survey respondents willing to participate who had current/previous experience of JAKi therapy, and invited to either take part in a telephone pilot interview or join one of three online focus groups. They were provided with Patient Information Sheets and Consent Forms (both co-designed with the two departmental Patient Experts). Written consent was obtained from all participants.

In December 2021, two of the 18 patients with IA took part in pilot interviews conducted by a Research Associate (AB) experienced in qualitative methods. No further amendments to the topic guide were required based on their feedback. The interviews (lasting up to 40 min) were audio-recorded, transcribed verbatim and their data were included in the final analysis. Focus groups were run in January 2022 using video-conferencing software, lasted up to 90 min and had five to six participants in each. They were facilitated by the Research Associate (AB) and a Patient Researcher (RW), audio-recorded and transcribed verbatim by the Research Associate. Transcripts were checked for correctness by RW, after which all the recordings were deleted. Patients were anonymised in all transcripts.

### Data analysis

Clinician and patient online survey data were exported from SurveyMonkey into SPSS Statistics 27 [IBM, Armonk, NY, USA] and NVivo 12 Pro [(QSR International, Doncaster, Vic, Australia] to aid descriptive statistical and thematic analyses. Patient interview and focus group transcripts were imported into NVivo 12 Pro to aid an inductive thematic analysis within a realist paradigm; whereby analysis was driven by patients’ accounts of their experiences, meaning and reality [20]. Codes were generated by the Research Associate (AB), cross-checked by Patient Researcher SdS, and themes and subthemes were identified by the Research Associate by looking for recurring patterns in the data [21]. These were further refined by SdS and agreed upon by the whole research team.

## Results

### Clinician survey

Fifty-one clinicians responded: 37 Consultants, 7 Registrars, 5 Clinical Nurse Specialists, 1 Clinical Fellow and 1 ‘other rheumatology role’ (not stated). Eleven of 12 UK regions were represented; with the highest number of respondents from Greater London (17.6%), North West England (15.7%) and East of England (13.7%). The proportion of clinicians working in secondary care was 68.6%, with the remaining 31.4% working in tertiary care. For full clinician demographics, see Additional file 4.

**Fig. 1:**
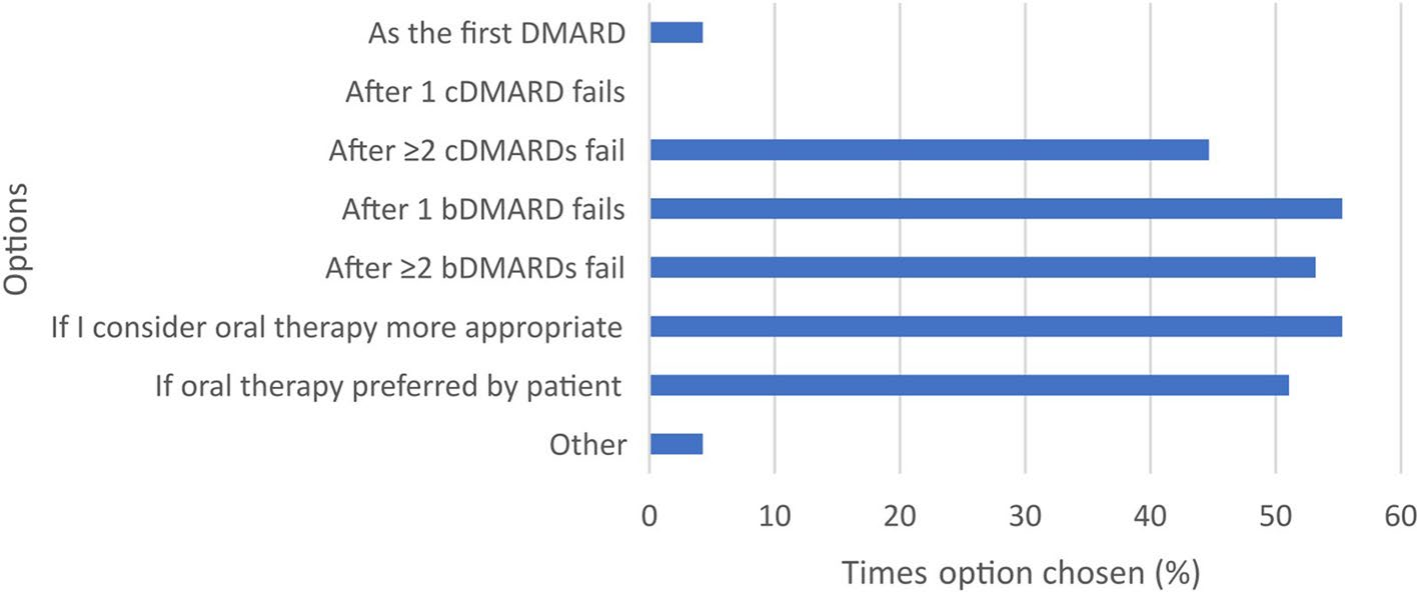
Point at which JAKi therapy usually started *(multiple response)*. b = biological; c = conventional; DMARD = disease-modifying antirheumatic drug

**Fig. 2:**
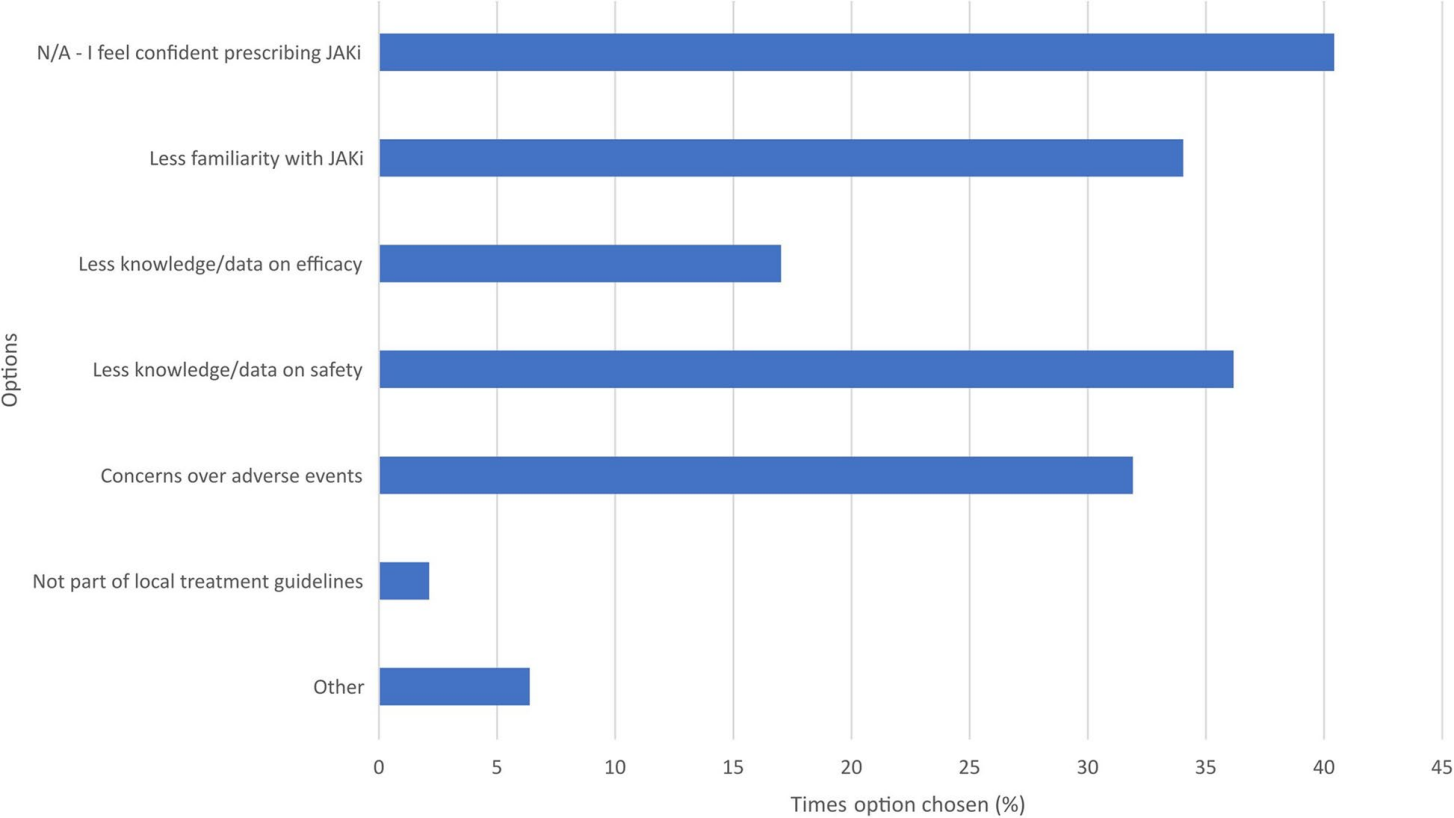
Reasons for feeling less confident in prescribing a JAKi compared with other advanced therapies *(multiple response)*. N/A = not applicable

Clinicians reported that their JAKi-naïve patients ask them about JAKi therapies never (39.2%), rarely (39.2%) or occasionally (21.6%). Ninety-six per cent of clinicians indicated prescribing JAKi in their clinical practice, 91.5% of whom prescribe as per their local guidelines. Figure 1 shows at what point in a patient’s treatment journey a JAKi is usually started; whilst Fig. 2 shows clinician confidence in prescribing JAKi, with possible reasons for lacking confidence.

JAKi were prescribed as a monotherapy frequently (72.3%), infrequently (25.5%) or never (2.1%).

**Fig. 3:**
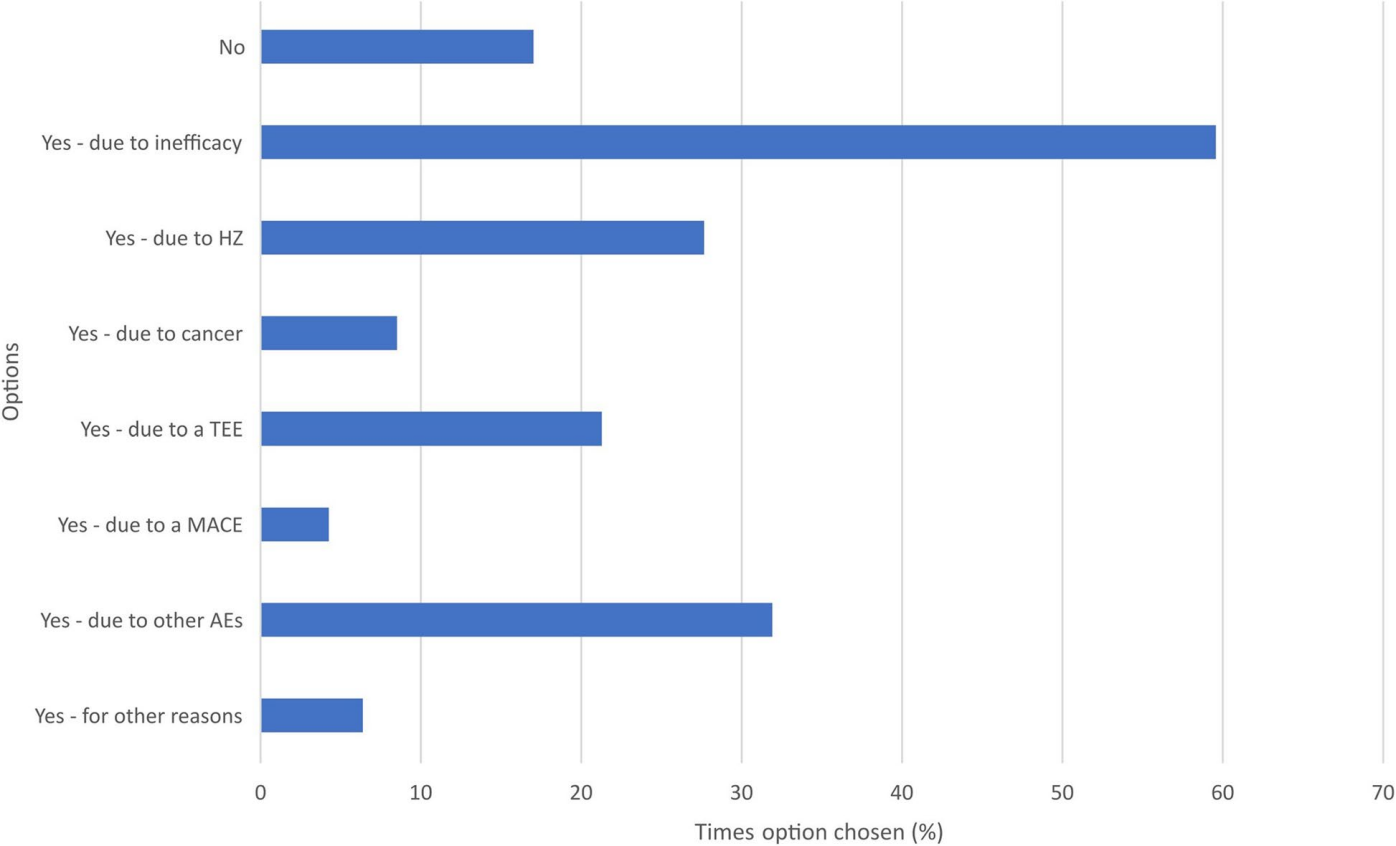
Discontinued JAKi in patients? *(multiple response)*. AEs = adverse events; HZ = herpes zoster; MACE = major adverse cardiovascular event; TEE = thromboembolic event

Figure 3 shows whether clinicians have had to discontinue JAKi in their patients and possible reasons why.

**Fig. 4:**
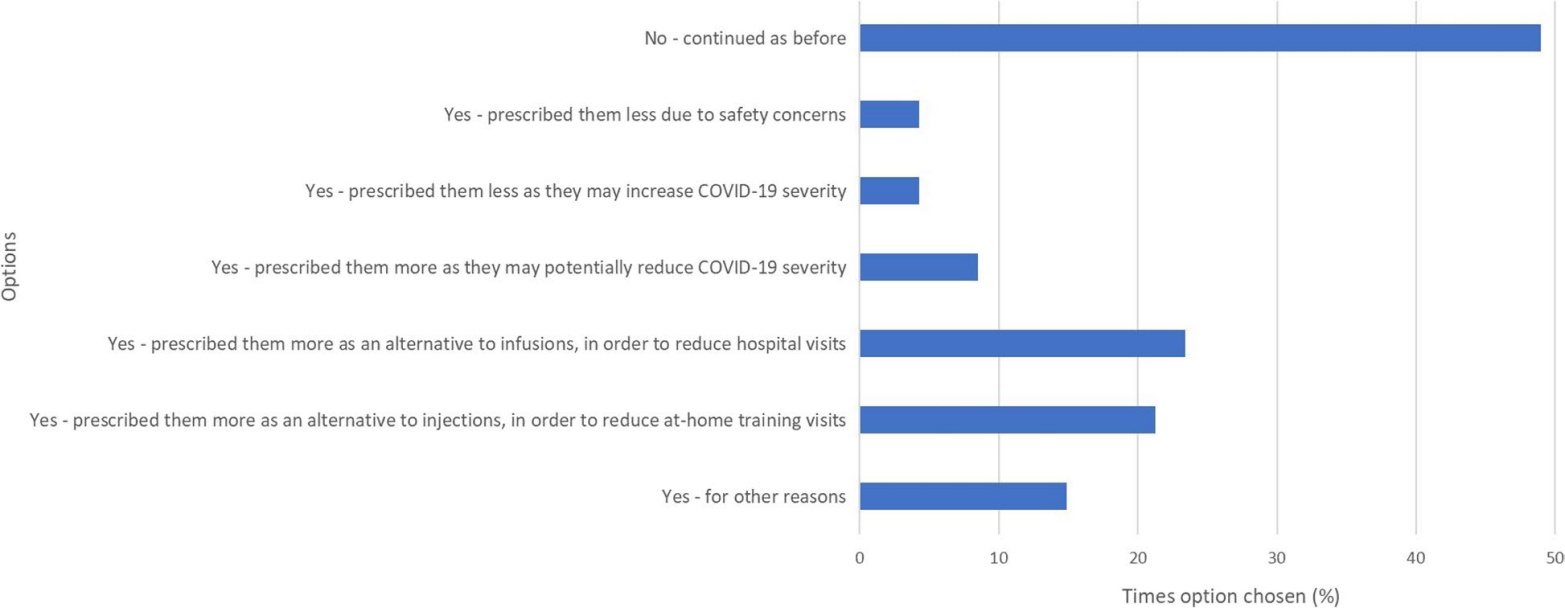
COVID-19 pandemic affected your JAKi prescription?

After discontinuation, 55.3% of clinicians would consider switching patients to another JAKi. Figure 4 shows whether the COVID-19 pandemic has affected the prescribing of JAKi and possible reasons why.

Seven free text responses were left for ‘other reason’; 6/7 highlighted the benefits of the shorter half-life of JAKi. Table 1 shows the free text responses received corresponding to the ‘other’ option in Figs. 1, 2, 3 and 4.

**Table 1:** Clinician survey free text responses for ‘other’ option in Figs. 1, 2, 3 and 4

**At what point in a patient’s treatment journey do you usually start JAKi therapy? **(Fig. 1)
*“Only in young patients without comorbidities or risk factors for VTE, and usually when options are limited or a patient is very adverse to injectable therapies.”* - Clinician 9 (Consultant, East of England) *“If perceived advantages for JAKi.”*—Clinician 35 (Consultant, Scotland)
**If you feel less confident in prescribing a JAKi, compared with other advanced therapies, why is this? **(Fig. 2)
*“Limited in PsA as upa not yet NICE approved and tofa needs MTX.”*—Clinician 23 (Consultant, South East England) *“Cost relative to biosimilars.”*—Clinician 35 (Consultant, Scotland) *“New advice for treating at lower levels of DAS28 hasn’t yet been approved in our region.”*—Clinician 46 (Consultant, Northern Ireland)
**Have you had to discontinue JAKi in your patients?** (Fig. 3)
*“Safety concerns in people over 65, people with co-morbidities.”*—Clinician 9 (Consultant, East of England) *“2 patients developed leg oedema which seemed to be linked [to the JAKi].”*—Clinician 26 (Consultant, Greater London) *“Patient planning pregnancy.”*—Clinician 35 (Consultant, Scotland)
**Has the COVID-19 pandemic affected your prescribing of JAKi? **(Fig. 4)
*“Not applicable—I did not prescribe JAKi prior to the pandemic as it occurred early in my training”*—Clinician 3 (Registrar, East of England) *“Yes—as a short half-life they were seen as being safer than bDMARDs, in respect of SARS-CoV2 (guidance from BSR).”*—Clinician 4 (Consultant, East of England) *“Initially I prescribed them for RA patients in preference to rituximab/tocilizumab as didn’t need day unit attendance, and felt safer for older patients as v short half-life. Started to question that when the Covid safety/ vaccine efficacy data came out, and serious doubts set in with the recent safety alerts.”*—Clinician 6 (Consultant, East of England) *“Shorter half life.”*—Clinician 28 (Consultant, North West England) *“Shorter duration of action so stop if infection.”*—Clinician 30 (Consultant, North West England) *“Prescribed more as quick on and quick off so can be discontinued quickly in event of severe infection.”*—Clinician 31 (Registrar, Greater London) *“Shorter half life”*—Clinician 43 (Registrar, Greater London)

*bDMARDs* biological disease-modifying antirheumatic drugs, *BSR* British Society for Rheumatology, *Covid* coronavirus disease, *DAS28* disease activity score in RA based on 28 joints, *JAKi* janus kinase inhibitor, *MTX* methotrexate, *NICE* National Institute for Health and Care Excellence, *PsA* psoriatic arthritis, *RA* rheumatoid arthritis, *SARS-CoV2* severe acute respiratory syndrome coronavirus 2, *tofa* tofacitinib, *upa* upadacitinib, *VTE* venous thromboembolism

Fourteen rheumatologists left additional comments at the end of the survey (see Additional file 5); nearly all were about JAKi safety (MACEs, malignancy and VTEs). Also mentioned were JAKi being an effective option in patients with disease resistant to other therapies, JAKi being prescribed in needle-phobic patients and the feeling that JAKi lose efficacy after a few years.

### Patient survey

One hundred and seventy-five surveys were received of which 141 (80.6%) were eligible for the final analysis; as 30 patients didn’t meet the inclusion criteria, two completed the survey twice and two surveys were incomplete. Patients were largely female (92.2%) and White (94.3%), with an age range of 25 to 78 years (M = 52.6, SD = 12.4). Most were diagnosed with RA (73.8%), and the remaining 26.2% with PsA. Disease duration ranged from one to 44 years (M = 14.0, SD = 10.5).

Sixty per cent of patients indicated they were on biologics: 48.2% by self-injection (etanercept the most common at 35.9%) and 11.3% by infusion (rituximab the most common at 47.1%); with 39.7% on a JAKi (60.7% baricitinib, 23.2% tofacitinib, 8.9% filgotinib, 7.1% upadacitinib). All 12 UK regions were represented; with most respondents from South East England (13.5%), South West England (12.1%) and Greater London (10.6%). See Additional file 6 for full patient demographic information.

**Fig. 5:**
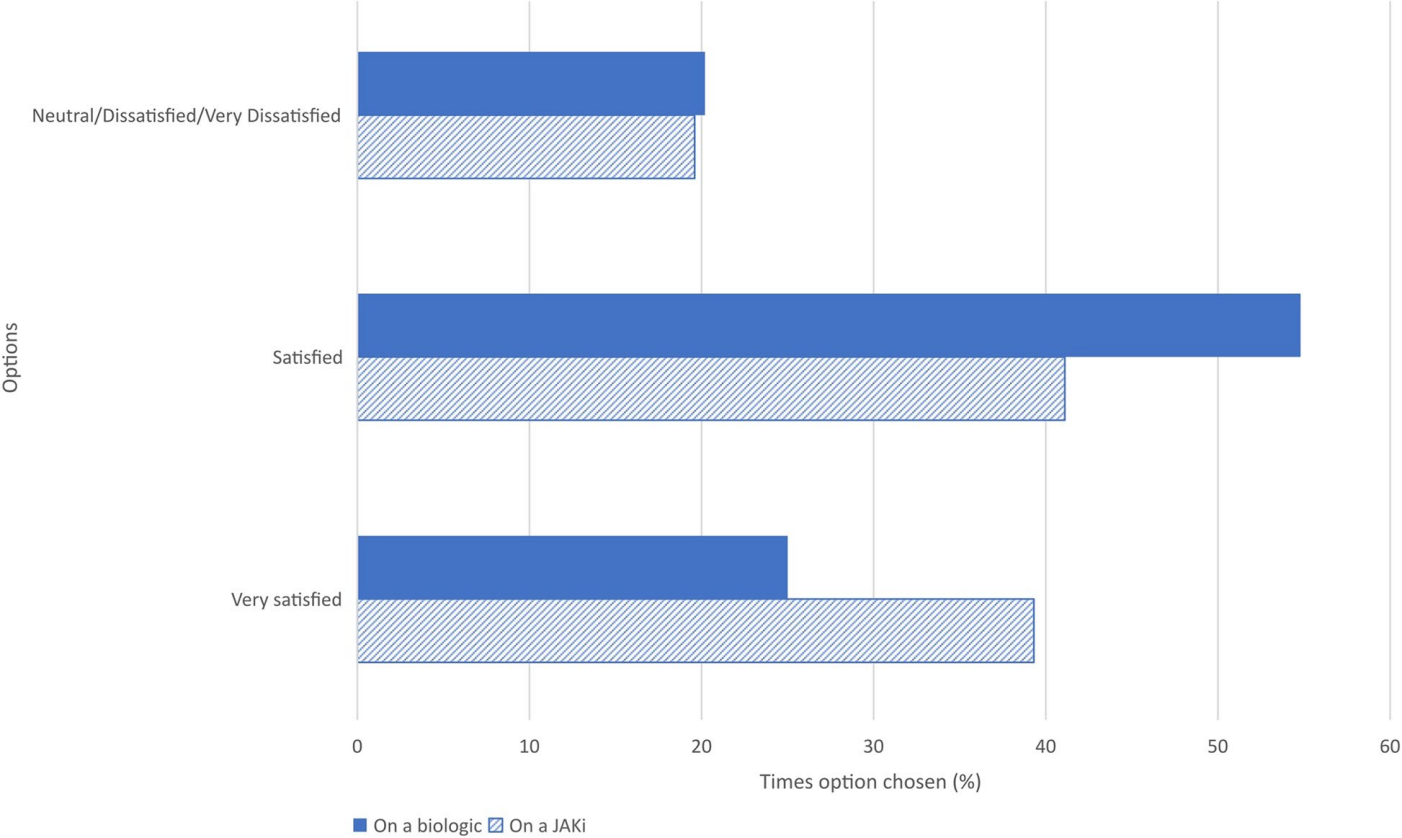
Overall satisfaction with arthritis medication

Overall medication satisfaction for patients on biologics versus those on JAKi is shown in Fig. 5.

Table 2 shows free text response analysis with regard to overall satisfaction with arthritis medication.

**Table 2:** Patient survey—overall satisfaction with current arthritis medication

**Themes**	**Illustrative quotes**
For patients currently on a biologic:	
*Benefits of biologics*	*“The biologics medication [etanercept] made a big difference to my inflammation/DAS [disease activity score]…*etc*. Better than methotrexate alone & couple of other meds [medications] that were tried.”*—Patient 70 (RA, East of England) *“With the arthritis side, the taltz [ixekizumab] worked immediately and I could go back to the gym…”*—Patient 113 (PsA, Greater London) *“Can tolerate abatacept without noticeable side effects. Seems to still work fine.”*—Patient 49 (RA, North East England)
*Drawbacks of biologics*	*“It [etanercept] generally keeps my condition under control but it is still active with continued joint deterioration or deformation.”*—Patient 45 (RA, Wales) *“I feel that it works but wears off [secukinumab] and injections are painful”*—Patient 131 (PsA, West Midlands) *“Due to COVID, not wanting to take a biologic [etanercept] that stays in my system more than a week.”*—Patient 123 (PsA, South West England)
For patients currently on a JAKi:	
*Journey onto JAKi*	*“I went through so many other drugs—tabs [tablets], injs [injections] & iv [intravenous] infusions. I had a lot of unpleasant side effects with them.”*—Patient 100 (RA, West Midlands) *“Had tried several biologics, had reaction to them all: rash, liver function.”*—Patient 107 (RA, Wales)
*Benefits of JAKi*	*“Since starting baricitinib…I feel better than I have done in several years. I can do normal day to day jobs with no joint pain. I can walk instead of being pushed about in a wheelchair. I've got my life back.”*—Patient 105 (RA, West Midlands) *“My PsA has been uncontrolled for a long time as I have failed 5 biologics and all DMARDs [disease-modifying antirheumatic drugs]. This [tofacitinib] has given me better relief than any others…”*—Patient 14 (PsA, Greater London) *“It [baricitinib] keeps my disease under control and I can stop it at short notice if I have an infection. It kicks in fast once I restart it. Also convenient to travel with as a tablet and no more injections (which I hated)!”*—Patient 85 (RA, Greater London)
*Drawbacks of JAKi*	*“Neutropenia and reasonable but not ideal effectiveness, plus lots of side effects [on tofacitinib].”*—Patient 7 (PsA, North East England) *“Baricitinib worked really well for me initially but in the last couple of months I feel it hasn't been quite as effective.”*—Patient 90 (RA, Scotland) *“Joints are good but I’ve just had a severe bout of shingles [on baricitinib].”*—Patient 91 (RA, South East England)

*JAKi* janus kinase inhibitor, *PsA* psoriatic arthritis, *RA* rheumatoid arthritis

Seventeen patients (12.1%) reported previously stopping a JAKi (nine tofacitinib, four baricitinib, one filgotinib, three unknown). Almost half (47.1%) of these patients discontinued their JAKi within three to six months of starting, 23.5% between six months to one year; 23.5% over one year, and 5.9% between one and three months. Free text analysis showed inefficacy (10 references) was the most common reason for stopping a JAKi, followed by non-MACE adverse effects (8 references) e.g. diarrhoea, vomiting, stomach pain, sore leg muscles, neutropenia, chest infections and pneumonia. One female with RA (aged 64) stopped her JAKi due to a heart attack. See Additional file 7 for full responses.

**Fig. 6:**
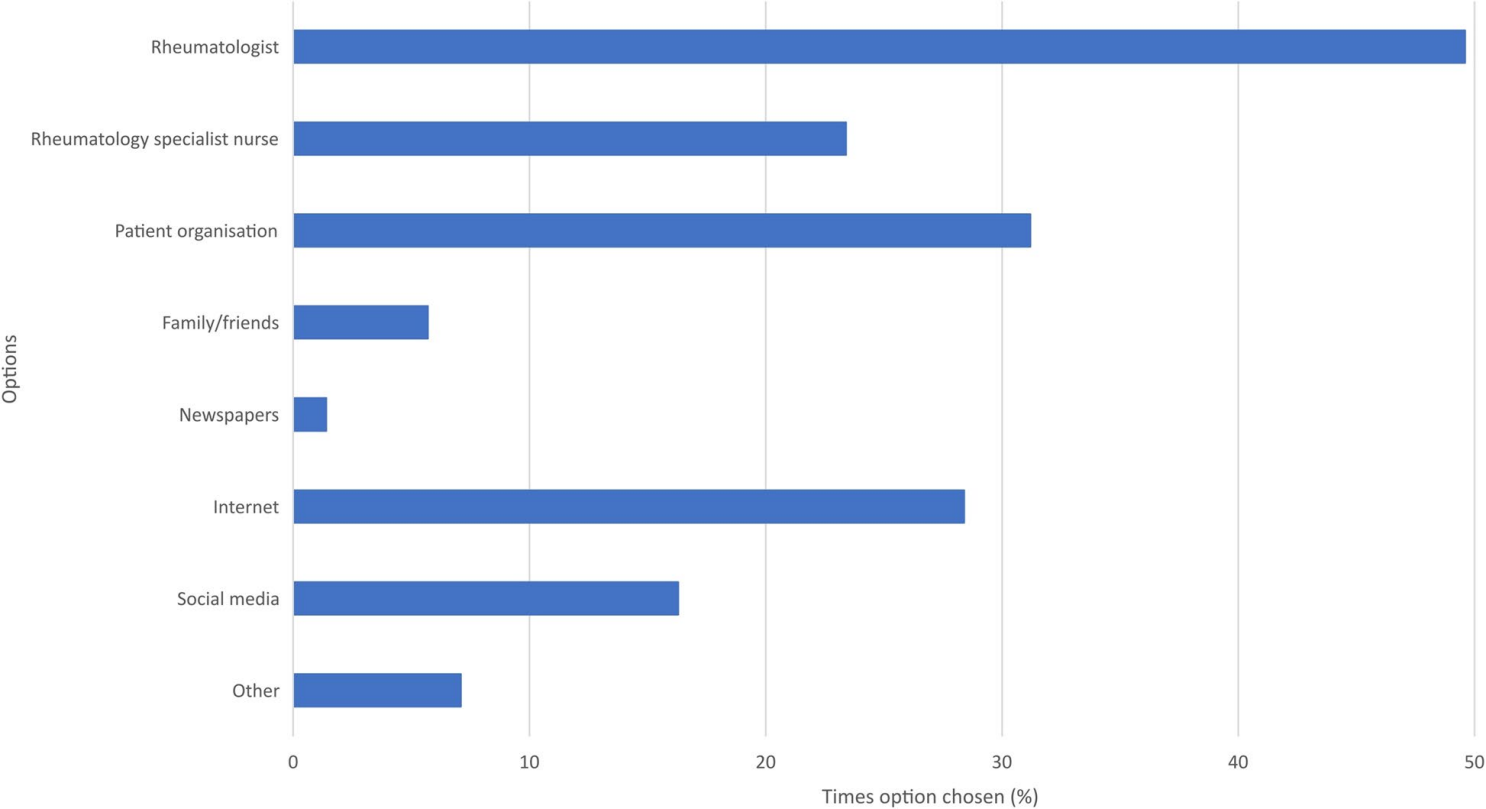
Sources from which patients heard about JAKi *(multiple response)*

When asked how aware they were of JAKi, patients chose: ‘somewhat aware’ (40.4%), ‘very aware’ (26.2%), ‘not aware at all’ (13.5%), ‘not aware’ (10.6%) and ‘not very aware’ (9.2%). Sources of information from which patients had heard about JAKi are shown in Fig. 6.

‘Other’ sources of information reported were: this survey (5), through working in an allied medical field (3), from peer-reviewed literature (1) and through a patient support group (1).

When asked whether they prefer an oral therapy (such as a JAKi) if currently on a biologic, patients chose ‘don’t know’ (45.2%), ‘yes’ (27.4%) and ‘no’ (14.3%). Six per cent chose the ‘Not applicable as already tried a JAK inhibitor’ option; whilst 7.1% of responses were invalid (patients who were not currently on a biologic, or had previously used a JAKi but did not choose the ‘Not applicable as already tried a JAK inhibitor’ answer option). Fifty-nine additional comments were left at the end of the survey - themes and illustrative quotes are in Table 3.

**Table 3:** Patient survey—additional comments

**Themes**	**Illustrative quotes**
For patients currently on a biologic:	
*Perceptions about biologics*	*“Cannot understand why something that has worked for years [etanercept], suddenly stops doing.”*—Patient 58 (RA, North West England) *“Worried about vaccine [COVID] effectiveness as on rituximab.”*—Patient 52 (RA, Greater London)
*Access to and knowledge about JAKi*	*“CCG [Clinical Commissioning Group in England] refused JAK inhibitor funding request and instead approved injected biologic…”*—Patient 59 (PsA, Greater London) *“Definitely more [patient] education needed on JAK inhibitors…I feel ignorant of their efficacy and potential.”*—Patient 128 (PsA, East of England)
*Concerns about JAKi*	*“I am concerned about the side effects of JAK inhibitors, I’ve seen that the FDA [U.S. Food and Drug Administration] is concerned about JAK inhibitors now.”*—Patient 32 (RA, North East England) *“Although I can appreciate JAK inhibitors are the next generation biologics, my concern is that as they are tablet form, what affect could they have on those like me who suffer from gut conditions too.”*—Patient 122 (PsA, North East England)
For patients currently on a JAKi:	
*Journey onto JAKi*	*“Filgotonib appears to be a new therapy, very little information prior to commencing medication even in the enclosed leaflet.”*—Patient 20 (RA, East Midlands) *“COVID implication meant I went onto JAK rather then Rituximab.”*—Patient 24 (RA, North West England)
*Benefits of JAKi*	*“The influence of JAK inhibitors in my life has not only had a big physical impact, but…decreased pain and the ability to take part in exercise has led to improved mental health.”*—Patient 12 (RA, Northern Ireland) *“The shorter half life with a JAK is a benefit, I have a surgery next week and the drug will wash out in 48 h which is helpful!”*—Patient 3 (RA, Greater London) *“…oral tablets are much more convenient, especially when away from home!”*—Patient 95 (RA, East of England)
*Drawbacks of JAKi*	*“I have been on a JAK inhibitor since March 2021 and I am at present in a major flare which might indicate the drug is not working.”*—Patient 21 (RA, West Midlands) *“I've gained weight on Xeljanz [tofacitinib]. I'd be interested to know if others have found this too.”*—Patient 89 (RA, North West England)

*COVID* coronavirus disease, *JAKi* janus kinase inhibitor, *PsA* psoriatic arthritis, *RA* rheumatoid arthritis

### Patient interviews and focus groups

Data from 18 patients were used for the thematic analysis: two patients from the pilot interviews and 16 from the three focus groups. Patients were largely female (83.3%) and all White (100%), with an age range of 28 to 71 years (M = 57.6, SD = 11.6). Fourteen patients had been diagnosed with RA, three with PsA, and one with RA and PsA. Disease duration ranged from four to 38 years (M = 17.0, SD = 10.0).

Seventeen of 18 patients were currently on a JAKi (seven tofacitinib, seven baricitinib, two filgotinib, one upadacitinib); mean time on a JAKi was three years (SD = 1.1). One patient was on a biologic after previous JAKi use. Of all patients, 61.1% had concurrent cDMARD and/or steroid usage, and 83.3% were previously on a biologic. Nine of 12 UK regions were represented; with most respondents from North West England (22.2%), South West England (16.7%) and Northern Ireland (16.7%). See Additional file 8 for full patient demographics.

Four main themes were identified from all the data (further illustrative quotes can be found in Additional files 9, 10, 11 and 12):

#### Theme 1: journey onto JAKi

##### Decision-making process

For many patients it was a shared decision to go on a JAKi and they felt well-informed by their rheumatology team, whilst some patients had done their own research. However, other patients reported a lack of information being given by their healthcare team or not being involved in the decision to try a JAKi. A minority of patients felt it was too much to decide and preferred their clinician to make the decision on which treatment was best for them.

##### Why JAKi prescribed

For nearly all patients JAKi were prescribed as a drug of last resort; having cycled through many other drugs previously which didn’t work for them and/or gave them adverse side effects:

*“I was just about on every different type of biologic…Some weren’t great and some had bad side effects, some had no effect whatsoever, and then…the JAKs came out…”*—Patient 6 (RA, South West England).

Other reasons given for being put on a JAKi, tofacitinib in both instances, were: i) it could treat both RA and PsA, and ii) its potential cost-effectiveness to the health service in the long run (in terms of saving hospital resources and JAKi becoming cheaper than biologics once off patent).

##### Expectations of JAKi

Expectations for most people starting JAKi therapy were low; they mainly spoke of having “hope”. Those in employment hoped the drug would help them stay in work:

*“I think my expectations were literally that I didn’t have many expectations. I hoped that [filgotinib] would get me to my thirties without having any more metal in me [from joint replacements]…I hoped that it would let me continue the job that I loved.”*—P7 (RA, Wales).

When there was expectation, it was that the JAKi would control their IA and possibly lead to remission. A few patients, however, were wary about potential adverse effects such as shingles (herpes zoster caused by reactivation of latent varicella-zoster virus).

##### Awareness of and access to JAKi

Whilst rheumatology clinicians know about JAKi, awareness /knowledge of JAKi from non-rheumatology health professionals seemed variable with general medical practitioners and community pharmacists knowing the least, and oncologists the most. A few patients raised concerns about lack of patient awareness of and earlier access to JAKi, feeling damage is being done to joints whilst prescription of these drugs is delayed by guidelines/protocols and rheumatologists’ reluctance to prescribe JAKi.

#### Theme 2: experience of using JAKi

##### Time to take effect

Many patients commented on how JAKi kick in quickly (days or weeks) bringing about disease control and with no need for bridging steroids, unlike with biologics. However, two patients said it took between three and eight months for the JAKi to have an effect on their IA.

##### Quality of life

Patients could see a multitude of benefits from taking a JAKi. Many patients reported being able to move more since being on a JAKi which in turn lifted their mood. Some patients even felt able to exercise, which they weren’t able to do before, which resulted in weight loss and improved health:

*“ …with more mobility you can exercise more, which helps you to lose weight. I have also lost weight since taking it [baricitinib]. So, it has a very effective knock-on effect for your lifestyle and for your health in general…”*—Patient 3 (RA, North West England).

Conversely, two patients said they had gained weight since being on the JAKi and had heard this happens to other patients too. Many patients reported having a better quality of life since being on a JAKi, which resulted in less dependence on others and confidence in being able to make plans without the worry of being too ill to follow through. Most patients who were still working at the time of starting a JAKi, mentioned how the JAKi has helped them to stay in work.

##### Comparison with previous treatments

When asked how JAKi compared to their previous arthritis treatments, patients described them as an *“absolute game changer”*, *“transformative”*, *“superior”*, and the difference being *“night and day”*.

Patients reported JAKi being convenient to take; as in tablet form they were especially good for people who don’t like needles and meant not having to take time out of your day to attend the hospital for infusions. One patient mentioned they sometimes struggle to remember to take their JAKi due to their work patterns, whilst another said they are doing so well on their JAKi that they forget they need to take it for their IA. Patients also found JAKi convenient for travelling both locally and abroad; as they no longer had to worry about having a fridge available, carrying a sharps bin, being questioned at the airport and having paperwork for their injections.

##### Side effects

Some patients had few to no side effects; whilst nearly two-thirds reported having raised cholesterol levels since starting their JAKi. As a consequence, some patients were prescribed statins:

*“Yes, I have got a cholesterol result that is going up. Each blood test that I have, it goes up slightly…but the GP was very quick to get me on to a standard anti-cholesterol tablet [statin].”*—P4 (RA, Northern Ireland).

One patient reported getting high blood pressure, frizzy hair, dry mouth and repeated urinary tract infections since starting their JAKi; whilst another said they have developed cognitive impairment (brain fog and memory issues). Both were on tofacitinib.

##### Stopping and switching JAKi

A few patients temporarily stopped their JAKi due to infections such as cold sores (oral herpes), urinary tract and chest infections. One patient temporarily stopped their JAKi due to a low number of neutrophils and then restarted at a lower dose; whilst another patient had their JAKi dose lowered due to a flare of their pre-existing genital herpes, but then raised again as the lower dose was unsuccessful in controlling their IA. Another patient had to permanently stop their JAKi due to neutropenia, recurrent infections and raised liver enzymes.

One female RA patient aged 70 years reported having their JAKi switched to baricitinib, as tofacitinib was giving her palpitations and atrial fibrillation; whilst another female RA patient aged 64 years was currently undergoing investigations for a heart problem which started after taking baricitinib.

#### Theme 3: Concerns over using JAKi

##### Safety concerns of patients

Some patients were concerned over possible increased risks of cardiovascular disease and cancer with JAKi. One patient specifically mentioned hearing about these increased risks with JAKi through warnings from the U.S. Food and Drug Administration (FDA) which caused him concern:

*“FDA had not given permission for it [tofacitinib] to be used for a while, a big drug administration in America…The tofacitinib has been found to have issues around cardiovascular events and cancer. So, sorry to say that, but that is possibly a class effect, who knows? So, that is a worry.”*—Patient 13 (RA, Scotland).

Other concerns about JAKi use mentioned were serious and opportunistic infections, whether it affects fertility and the risk of liver failure. One patient felt that although JAKi are newer drugs and there is not enough long-term data on them, they were not concerned by what they have read so far.

##### Safety concerns of health professionals

One patient described their pharmacist from the GP surgery and orthopaedic surgeon having concerns about them being on a JAKi. Another patient was asked by their rheumatology consultant to stop taking their JAKi due to concerns over the ORAL Surveillance study data, whilst a third patient mentioned rheumatologists in Northern Ireland switching patients from tofacitinib to baricitinib due to concerns raised by this study.

##### Patient attitudes towards JAKi use

Despite having some concerns over JAKi use, the majority of patients thought that the benefits outweighed the risks and would rather have a better quality of life now than worry about what may happen in the future:

*“…I think the benefits [of filgotinib] outweigh the risks…Life is too short to be worrying about in 10 years, 20 years, or 30 years’ time. I would rather enjoy my next couple of years…”*—Patient 7 (RA, Wales).

One patient was less concerned; believing JAKi overall are safe and have associated risks just like other medications. Long-term, a few patients were worried as to whether the JAKi will eventually stop working for them.

#### Theme 4: JAKi and the COVID-19 pandemic

##### Attitudes towards JAKi during the pandemic

Some patients were more worried about catching COVID-19 whilst on an immunosuppressant, such as a JAKi, which affected their behaviour during the pandemic; causing them to limit their activities and avoiding meeting up with people:

*“I think it has been in the news quite a bit; people that are immunosuppressed and the problems that you have with COVID, and that has really impacted on me and has made me think more about…the things that I do. But that would have had been the same no matter what drug you were on that suppressed your immunity.”*—Patient 8 (RA, Northern Ireland).

Whereas one patient was too anxious to stay on the JAKi, because of public messaging on increased susceptibility to COVID-19, and therefore stopped taking it for a while. A few patients mentioned their disease control was more of a concern than worrying about catching COVID-19 so they continued taking their JAKi. Also, a few patients mentioned temporarily stopping their JAKi because they were going to have a COVID-19 vaccination, or due to having COVID-19 itself.

##### Starting a JAKi during the pandemic

Two patients reported starting a JAKi during the pandemic: one so that they did not have to leave the house and risk infection exposure, and the other because rituximab was found to impair antibody production in response to COVID-19 vaccines.

##### Potential benefits of JAKi during the pandemic

One patient was happier to be a on a JAKi rather than a biologic during the pandemic; due to its shorter half-life and therefore ability to quickly reverse immunosuppression. Some patients had also heard that JAKi were being used to treat patients with moderate to severe COVID-19 and were glad to already be on these drugs, believing they may confer some benefit:

*“I discovered that they [clinicians] were using it [JAKi] to treat COVID and my ears pricked up when this came on the news once…they [clinicians] were using it as one of an array of drugs to reduce the symptoms of COVID and I thought that is a bonus [being on a JAKi].”*—Patient 1 (PsA, Greater London).

## Discussion

This study revealed general awareness of JAKi, with variable degrees of knowledge amongst IA patients and non-rheumatology health professionals. This is an area which requires focus; not only to increase awareness of this newer and promising class of drugs amongst all patients with IA, but also within the wider health professional community. Alongside increased sharing of JAKi prescribing experience and knowledge within rheumatology teams and research to help clinicians identify/predict which patients might be most suited and responsive to JAKi therapy.

Ninety-one per cent of prescribing clinicians followed their local prescribing guidelines, with many indicating they prescribe JAKi after the failure of two or more conventional DMARDs (cDMARDs) or one or more bDMARDs. Many also said they would prescribe JAKi if they thought oral therapy was more appropriate and/or their patient preferred it. Almost three-quarters of clinicians prescribed JAKi as a monotherapy. This was slightly higher than the 57% rate found by Taylor et al. for RA patients across six Western European countries (UK included) [22], although we acknowledge that direct comparisons are inappropriate in view of the differences in the data used. Some clinicians and patients reported having concerns about JAKi increasing the risk of cardiovascular disease, malignancy and VTEs; some specifically mentioning or alluding to the ORAL Surveillance study results [11] and subsequent regulatory authority warnings [13, 14] as a trigger for these concerns. The ORAL Surveillance study has been one of the most discussed studies in the context of JAKi and has raised important concerns that have kept the clinical and academic rheumatology communities in active debate.

When clinicians permanently discontinued JAKi, it was primarily due to inefficacy and non-MACE adverse effects (including herpes zoster and VTEs). Similarly, patients most commonly reported discontinuation due to inefficacy closely followed by non-MACE adverse effects, with nearly half of discontinuations within three to six months. Recent real-world studies also found JAKi primarily being stopped in IA patients due to inefficacy and adverse events [23, 24], though conversely the JAK-pot collaboration (combining data from 19 registries, mainly European) found JAKi discontinuation more likely to occur due to adverse events than inefficacy [25]. Fifty-five per cent of clinicians would consider switching patients to another JAKi after discontinuation. This is in line with recommendation 10 of the 2022 update of the EULAR Recommendations for the Management of Rheumatoid Arthritis with Synthetic and Biological Disease-modifying Antirheumatic Drugs which allows for switching to another tsDMARD after initial failure [26].

Some patients believed there to be challenges with getting earlier access to JAKi due to restrictive guidelines, funding restraints and clinician reluctance to prescribe. Current National Institute for Health and Care Excellence guidelines say JAKi can only be prescribed for IA patients if they have responded inadequately to or cannot have other DMARDs, including at least one biologic [27–33]. Many patients in the focus groups were prescribed their JAKi as part of shared decision-making with their clinician and nearly all were prescribed it as a drug of last resort, having cycled through multiple previous therapies. This resulted in expectations of taking a JAKi being low and patients just having “hope” the drug would work for them. Data from the British Society for Rheumatology Biologics Register for Rheumatoid Arthritis confirms that the majority of patients prescribed JAKi in the UK, up until April 2019, had had a median of three bDMARDs previously [34].

The most common side effect of JAKi reported by interview/focus group patients was a raised cholesterol level, which had resulted in some being put on a statin. Hypercholesterolaemia is a known side effect of JAKi use [6]. Temporary discontinuation of JAKi often took place when patients had an infection. Three patients (all elderly females with RA) had experienced heart problems since starting a JAKi. The 2022 EULAR Recommendations update proposes that if cDMARDs alone cannot achieve the treatment target, ‘JAK inhibitors may be considered’ taking into account pertinent risk factors defined as ‘age over 65 years, history of current or past smoking, other cardiovascular risk factors, other risk factors for malignancy, and risk factors for thromboembolic events’ [26]. The EMA also updated their recommendations in late 2022 to caution against the use of JAKi in IA patients aged 65 years or above, at increased risk of MACEs, current or previous smokers who smoked for a long time, people at increased risk of cancer and those with a VTE risk, unless there was no suitable alternative treatment available [35].

Despite some patients experiencing side effects and knowing of possible increased risks of MACEs and cancer, most patients felt the benefits of being on a JAKi outweighed the risks for them; with many having an attitude of it being preferable to be able to enjoy life now rather than worrying about what may happen down the line. This was in contrast to clinicians’ more risk-averse approach to JAKi prescription. This demonstrates the importance of educating patients about JAKi so they are informed and making shared decisions in consultations; balancing risks and benefits, and taking into account patient preferences. This has been underpinned by Overarching Principle A in the 2022 EULAR recommendations update: ‘Treatment of patients with RA should aim at the best care and must be based on a shared decision between the patient and the rheumatologist’ [26].

It is important for clinicians to have one-to-one discussions with patients who are suitable for JAKi therapies regarding their benefits and risks. We appreciate that rheumatologists are under considerable time pressure during clinics so suggest they provide a brief overview and then signpost patients to reliable sources of information on JAKi, e.g. patient charity publications. A longer follow-up appointment can then be booked with another member of the multidisciplinary team, e.g. rheumatology nurse or hospital pharmacist, who can discuss JAKi in more detail and respond to any questions patients may have. In the future, formal ‘decision aids’ may need to be developed.

Just over a quarter of patients on biologics had a preference for oral therapy, whilst almost half were undecided. Patients on JAKi were more likely to be ‘very satisfied’ overall with their arthritis medication than those on biologics. Many patients commented on how JAKi kicked in quickly (within days and weeks) and enabled them to move more and exercise, resulting in greater independence and a better quality of life. Most patients who were still working at the time of starting the JAKi, reported the JAKi had enabled them to remain in employment. Patients preferred the convenience of taking a tablet rather than self-injecting or having infusions, which required additional time spent at the hospital. Patients also found JAKi convenient for travel, versus injectable biologics. Both patients and clinicians agreed JAKi were beneficial for people who dislike needles/injections. In a recent study in PsA patients, Ogdie et al. also found a preference for oral therapy; top cited reasons being convenience to take, easier to travel with, easier to remember and having a dislike of needles/injections [36].

This study specifically explored perceptions of JAKi also in the context of the COVID-19 pandemic. Some patients worried about being on a JAKi during the pandemic, and thus altered their social behaviour, but highlighted that this would be the same no matter which immunosuppressant they were on. Glintborg et al. also found IA patients had more anxiety and practiced more self-isolating behaviours during the height of the pandemic [37]. However, overall patients in this study felt it was better to continue taking their JAKi and risk catching coronavirus than to have uncontrolled IA. A few patients did temporarily stop their JAKi due to having COVID-19 or after having their COVID-19 vaccine, in the hope they would then produce a better antibody response. Some patients were glad to be on a JAKi already, after hearing they were being used to treat patients hospitalised with COVID-19 [10], believing that this may confer some protection from the disease.

Almost half of clinicians reported that the COVID-19 pandemic had not affected their prescribing of JAKi. The majority of those who had changed their prescribing patterns indicated prescribing JAKi more as an alternative to biologics; in order to reduce hospital infusion and at-home injection training visits, and because JAKi have a short half-life which was seen as advantageous if patients caught the coronavirus. Patients also concurred with this saying they had been offered a JAKi to avoid them having to leave the house and expose themselves to possible infection, and that they felt much more comfortable taking a drug with a shorter half-life as it would be out of their system quicker should they catch infections. Indeed, British Society for Rheumatology guidance during the height of the pandemic was to use short-acting drugs (such as JAKi) when escalating RA treatment [38].

Limitations of this study include the small number of clinicians completing the survey, preventing meaningful statistical analyses; and that patient participants were mainly older, female and white, and therefore not truly representative of the UK IA patient population. Furthermore, data was not collected on pre-existing health conditions which may have affected patient response to previous treatments and disease activity was not reassessed at the time of the study. However, for the latter, patients would need to be at least in moderate disease activity states to be on a biologic or JAKi.

Only one patient in the qualitative part of the study had permanently discontinued a JAKi, therefore results may be overly positive for JAKi use. Most patients in the focus groups had previous failure of multiple biologics, which may have contributed to them having greater willingness to accept potential long-term risks from JAKi; and the surveys, interviews/focus groups had some retrospective questions which introduces an element of recall bias into the data [39].

Patient Researchers with IA co-facilitated the focus groups and co-analysed data which could have introduced an element of bias and influenced the results, however, the Research Associate led the focus groups and carried out primary data analysis which should have negated any potential patient bias. Both clinician and patient recruitment were hampered by the COVID-19 pandemic, as each group had other important priorities to contend with.

However, the study also has many strengths which include: being co-designed with patients and clinicians; the surveys were anonymous enabling honest responses; rheumatology clinicians who responded were mainly consultants (most likely to prescribe) and represented 11/12 UK regions; patients who completed the survey represented all 12 regions of the UK; the patient survey and interviews/focus groups included people on all 4 JAKi currently licensed in the UK; the patient sample was broadly in line with the UK population in terms of the proportion of patients with RA and PsA; and Patient Researchers were involved with co-facilitating the focus groups and analysing the data.

Since data were collected for this study, more studies have been published and are being conducted looking at whether the ORAL Surveillance study [11] results are borne out in real-world data and whether the increased risks of MACEs and cancer are applicable to all JAKi [40]. Also, updated guidance has since been released by EULAR and the EMA regarding which patient populations are at higher risk when prescribed a JAKi [26, 35]. Therefore, in the future, it would be useful to repeat this study to see how clinical practice and patient perception of JAKi have changed.

## Conclusions

Most patients have at least some awareness of JAKi, mainly from their rheumatology team. Clinicians prescribe JAKi frequently as a monotherapy and commonly after multiple other therapies have failed, with good results for patients. JAKi discontinuation is mainly due to inefficacy and adverse events (excluding MACEs). Some clinicians have concerns over JAKi safety; however, despite experiencing side effects and knowing of possible long-term risks, patients feel the benefits of JAKi outweigh the risks and are more likely to be ‘very satisfied’ on JAKi than biologics. Both clinicians and patients appreciate the short half-life of JAKi when it comes to infections, and patients like their convenience as an oral therapy. The COVID-19 pandemic resulted in increased prescription of JAKi as an alternative to infusions and injections, primarily to avoid potentially exposing patients to the coronavirus. Our findings highlight the importance of ensuring an informed shared decision-making process between a patient and their treating clinician when starting a JAKi; discussing potential risks and benefits, and taking into account individual patient circumstances and preferences.

### Supplementary Information

**Additional file 1. **Clinician Survey

**Additional file 2. **Patient Survey

**Additional file 3. **Patient interview and focus group topic guide

**Additional file 4. **Clinician survey demographics

**Additional file 5. **Clinician survey additional comments

**Additional file 6. **Patient survey demographics

**Additional file 7. **Patient survey - Why did you stop taking the JAK inhibitor?

**Additional file 8. **Patient interview and focus group demographics

**Additional file 9. **Theme 1: Journey onto JAKi

**Additional file 10. **Theme 2: Experience of using JAKi

**Additional file 11. **Theme 3: Concerns over using JAKi

**Additional file 12. **Theme 4: JAKi and the COVID-19 pandemic

## Data Availability

The datasets analysed during the current study are available from the corresponding author on reasonable request.

## Abbreviations

Anti-TNF: Anti-tumour necrosis factor
bDMARDs: Biological disease-modifying antirheumatic drugs
cDMARDs: Conventional disease-modifying antirheumatic drugs
COVID-19: Coronavirus disease 2019
DMARDs: Disease-modifying antirheumatic drugs
EMA: European Medicines Agency
EULAR: European Alliance of Associations for Rheumatology
FDA: Food and Drug Administration
IA: Inflammatory arthritis
JAKi: Janus kinase inhibitors
JAK-pot: JAK Inhibitors and Predictors of Outcome in rheumaToid Arthritis
M: Mean
MACEs: Major adverse cardiovascular events
MTX: Methotrexate
MHRA: Medical Health and Regulatory Authority
ORAL Surveillance: Oral Rheumatoid Arthritis Trial Surveillance
PsA: Psoriatic arthritis
RA: Rheumatoid arthritis
SARS-CoV2: Severe acute respiratory syndrome coronavirus 2
SD: Standard deviation
tsDMARDs: Targeted synthetic disease-modifying antirheumatic drugs
VTEs: Venous thromboembolisms
UK: United Kingdom
US: United States
