# Supplementary material for: Clinician and patient views on janus kinase inhibitors in the treatment of inflammatory arthritis: a mixed methods study
Source: BMC Rheumatol. 2024 Jan 17;8:1. doi: 10.1186/s41927-023-00370-7 (PMC10792861; doi:10.1186/s41927-023-00370-7)
Supplement: Supplementary file 1 — Additional file 1. Clinician Survey [file 41927_2023_370_MOESM1_ESM.docx]

**CLINICIAN views on janus kinase inhibitors in the treatment of inflammatory arthritis**

As part of a larger study, researchers from King’s College Hospital NHS Foundation Trust (London) are asking rheumatology clinicians to fill out a short online survey on the topic of janus kinase inhibitors (JAKi) in the treatment of inflammatory arthritis (IA). Our study has been reviewed and given favourable opinion by HSC REC A Research Ethics Committee. It has also been approved by the Health Research Authority.

To complete this 5-minute survey, you must be:

1. A rheumatologist or rheumatology nurse specialist
2. Practice in the UK 3. Regularly see patients with rheumatoid arthritis (RA) and/or psoriatic arthritis (PsA)

You are reminded that participation is entirely voluntary, and you do not have to complete the survey if you do not want to. Please be assured that any data you provide will remain strictly confidential. We will keep all information about you safe and secure. Once submitted, we are unable to remove your data from the study as we will not be able to identify your responses.

Upon completion of the survey you will be asked if you wish to provide an email address for any of the following purposes:

1. To receive a summary of the study results (expected Summer 2023)
2. To be entered into a prize draw to win one of 3 x £100 Love2Shop digital gift cards

Your email address will not be used for any other purposes or shared outside of the research team.

Researcher contact details:

Name: Dr Andrew Bassett (Research Associate)

Email: andrew.m.bassett@kcl.ac.uk

1. What is your role?


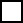
 Rheumatology Consultant

Rheumatology Registrar


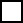


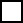
 Rheumatology Clinical Nurse Specialist


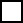
 Other rheumatology role (please state):…

1. In which UK region do you practice?

England


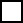
 North East
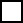
 Wales


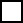
 North West
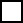
 Scotland


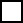
 Yorkshire and the Humber
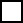
 Northern Ireland


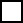
 West Midlands


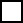
 East Midlands


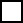
 South West


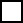
 South East


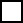
 East of England

Greater London


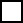


1. In which setting do you work?


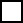
 Secondary care


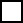
 Tertiary care


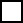
 Other (please state): ….

1. How much time do you spend doing research as part of your job role?


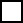
 0%


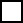
 1-25%


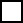
 26-50%


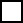
 >50%

1. Roughly what proportion of your patients with RA or PsA take a JAKi?


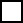
 <1%


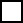
 1-5%


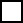
 6-10%

11-15%


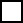


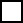
 16-20%


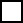
 >20%

1. How often do JAKi-naïve patients ask you about JAKi therapies?


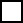
 Never


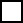
 Rarely


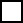
 Often


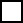
 Very often

1. Do you prescribe JAKi in your clinical practice? [If no, go to question 15]


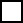
 Yes


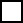
 No

1. Do you prescribe JAKi as per a local hospital or Clinical Commissioning Group pathway, Health Boards or Local Commissioning Group?


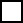
 Yes


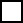
 No

9. At what point in a patient’s treatment journey do you usually start JAKi therapy? (choose all

that apply)

As the first disease-modifying antirheumatic drug (DMARD)


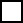


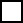
 After one conventional DMARD fails

After two or more conventional DMARDs fail

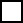
 After one biologic DMARD fails

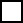
 After two or more biologic DMARDs fail


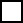


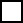
 If I consider oral therapy is more appropriate


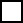
 If oral therapy is preferred by the patient


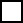
 Other (please state): ….

10. Do you prescribe JAKi as a monotherapy?


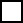
 Always

Frequently


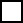


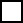
 Infrequently


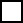
 Never

11. If you feel less confident in prescribing a JAKi, compared with other advanced therapies, why is this? (choose all that apply)


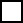
 N/A – I feel confident prescribing a JAKi


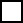
 Less familiarity with the use of JAKi


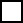
 Less knowledge/data on efficacy

Less knowledge/data on safety


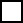


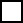
 Concerns over adverse events


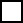
 Not part of our local treatment guidelines

Other (please state): ….


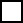


12. Have you had to discontinue JAKi in your patients? (choose all that apply)

- No
- Yes – due to inefficacy

Yes - due to herpes zoster infection


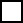


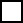
 Yes - due to cancer

Yes - due to a thromboembolic event


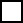


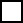
 Yes - due to a major adverse cardiovascular event


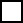
 Yes – due to other adverse events (please state): ……

Yes – for other reasons (please state): ……


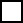


13. After discontinuation of a JAKi, would you consider switching patients to another JAKi?

- Yes
- No

14. Has the Covid-19 pandemic affected your prescribing of JAKi? (choose all that apply)


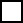
 No – I have continued as before


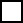
 Yes – I have prescribed them less due to safety concerns

Yes – I have prescribed them less as they may increase COVID-19 severity


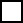


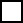
 Yes – I have prescribed them more as they may potentially reduce COVID-19 severity

Yes – I have prescribed them more as an alternative to infusions, in order to reduce hospital


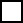


visits


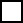
 Yes – I have prescribed them more as an alternative to injections, in order to reduce at-

home training visits


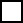
 Yes – Other reason (please state): ….

15. Any other thoughts you would like to share from your experience and/or knowledge on the use of JAKi in patients with IA? ….. (optional)

**Further contact (Optional)**

Please leave your email address if you wish to be contacted for any of the purposes below (select all that apply):


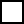
 To receive a summary of the study results (expected Spring 2023)


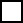
 To be entered into a prize draw to win one of 3 x £100 Love2Shop digital gift cards

Thank you so much for your input and time.

**Please click ‘submit’ to complete survey.**
