# Supplementary material for: Clinician and patient views on janus kinase inhibitors in the treatment of inflammatory arthritis: a mixed methods study
Source: BMC Rheumatol. 2024 Jan 17;8:1. doi: 10.1186/s41927-023-00370-7 (PMC10792861; doi:10.1186/s41927-023-00370-7)
Supplement: Supplementary file 2 — Additional file 2. Patient Survey [file 41927_2023_370_MOESM2_ESM.docx]

**Patient Views on janus kinase inhibitors in the treatment of inflammatory arthritis**

As part of a larger study, researchers from King's College Hospital NHS Foundation Trust (London) are asking patients with inflammatory arthritis to fill out a short survey on the topic of janus kinase inhibitors (JAK inhibitors). These are a new class of oral drugs which currently can be used to treat rheumatoid arthritis (RA) or psoriatic arthritis (PsA). The survey should take no longer than 10 minutes to complete.

Patients must meet the following criteria:

1. Aged 18 years or older

2. Live in the UK

3. Have been diagnosed by a rheumatologist with RA or PsA

4. Are currently taking a biologic (for 6 months or more) or JAK inhibitor (for 6 months or more) OR have previously taken a JAK inhibitor

The survey has been co-designed with patients and is split into 3 sections:

1. About you (demographic data collection)

2. About your condition and treatment

3. Awareness of JAK inhibitors

You are reminded that participation is entirely voluntary, and you do not have to complete the survey if you do not want to. If you are happy to complete the survey, please be assured that any data you provide will remain strictly confidential. We will keep all information about you safe and secure. Once submitted, we are unable to remove your data from the study as we will not be able to identify your responses.

At the end of the survey, please provide an email address if you wish one or more of the following:

1. To receive a summary of the study results (expected Summer 2023)

2. To be entered into a prize draw to win one of 3 x £100 Love2Shop digital gift cards

3. To participate in a 1.5 hours online patient focus group on your views and experiences of JAK inhibitors (payment of a £40 Love2Shop digital gift card after focus group, only patients who have current or previous use of JAK inhibitors are eligible for this part of the study)

Your email address will not be used for any other purposes or shared outside of the research team. This study has been reviewed and given favourable opinion by HSC REC A Research Ethics Committee. It has also been approved by the Health Research Authority.

If you need help with completing the survey, please email us using the details below and a member of the research team will contact you to assist.

Researcher contact details:

Name: Dr Andrew Bassett (Research Associate)

Email: andrew.m.bassett@kcl.ac.uk

**About you**

1. What is your age (years)? ………………
2. What is your gender?

Man Non-binary

Woman Other (please describe) …………………………

1. What is your ethnicity?

**White**

British

Irish

Romani or Irish traveller

Any other White background (please describe) ……

**Black or Black British**

Caribbean

African

Any other Black background (please describe) …………………………………..

**Asian or Asian British**

Indian

Pakistani

Bangladeshi

Chinese

Any other Asian background (please describe) …………………………………..

**Mixed**

White and Black African

White and Black Caribbean

White and Asian

Any other mixed background (please describe) ………………………………….

**Other ethnic group**

Arab


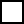
 Any other ethnic group (please describe) …………………………………………

1. Which UK region do you live in?

England


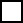
 North East
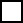
 Wales


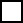
 North West
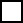
 Scotland


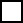
 Yorkshire and the Humber
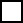
 Northern Ireland


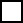
 West Midlands


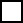
 East Midlands


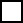
 South West


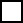
 South East


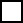
 East of England


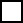
 Greater London

1. What is your current employment status?

Employed full-time Employed part-time

Self-employed Unemployed

Student Retired

Other (please describe): …………………………………………………………….

………………………………………………………………………………………….

1. What is your job type?


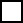
 Manual e.g. builder, plumber, factory worker


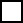
 Non-manual e.g. desk job, salesperson, teacher


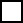
 Other (please describe): …………………………………………………………….


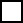
 Not applicable - I do not work

**About your condition and treatment**

7. Which condition have you been diagnosed by a rheumatologist with?

Rheumatoid arthritis

Psoriatic arthritis

8. What year were you diagnosed with rheumatoid arthritis or psoriatic arthritis?

………..

9. Which medication/s are you currently taking to treat your arthritis?


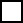
 JAK inhibitor (i.e. tofacitinib/Xeljanz®, baricitinib/Olumiant®, upadacitinib/Rinvoq® or filgotinib/Jyseleca®)


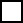
 Biologic by self-injection


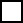
 Biologic by infusion


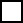
 Methotrexate in tablet form


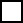
 Methotrexate as an injection


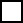
 Sulfasalazine (Azulfidine®)


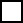
 Hydroxychloroquine (Plaquenil®)


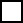
 Leflunomide (Arava®)


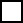
 Other

1. If you take a JAK inhibitor (i.e. tofacitinib/Xeljanz®, baricitinib/Olumiant®, upadacitinib/Rinvoq® or filgotinib/Jyseleca®), please state its name and the year that you started it.

Name: …….

Year: …….

1. If you take a Biologic by self-injection, please state its name and the year that you started it.

Name: …….

Year: …….

1. If you take a Biologic by infusion, please state its name and the year that you started it.

Name: …….

Year: …….

1. If you take another medication for your arthritis, please state its name and the year that you started it.

Name: …….

Year: …….

Medication examples include: Prednisolone, diclofenac, naproxen, abatacept (Orencia®), rituximab (Rituxan®), tocilizumab (Actemra®), sarilumab (Kevzara®), anakinra (Kineret®), adalimumab (Humira®), etanercept (Enbrel®), infliximab (Remicade®), certolizumab pegol (Cimzia®), golimumab (Simponi®), ixekizumab (Taltz®), secukinumab (Cosentyx®), ustekinumab (Stelara®), guselkumab (Tremfya®), gold injections (Myocrisin®), auranofin (Ridaura®), azathioprine (Imuran®, Azasan®), cyclosporine (Gengraf®, Neoral®, Sandimmune®) and apremilast (Otezla®)

14. How satisfied are you overall with your current arthritis medication/s?

Very satisfied

Somewhat satisfied

Neutral

Dissatisfied

Very dissatisfied

Reason for choice: ..…………………………………………………………………

……………………………………………………………………….....................… ………………………………………………………………….................................

15. Were you previously prescribed a JAK inhibitor and had to stop taking it?

Yes

No

1. Which JAK inhibitor did you take?

Tofacitinib

Baricitinib

Upadacitinib

Filgotinib

Don’t know/don’t wish to disclose

1. How long did you take the JAK inhibitor for?

Up to one month

One month to three months

From three months to six months

From six months to a year

Over one year

1. Why did you stop taking the JAK inhibitor?

………………………………………………………………………………………….

………………………………………………………………………………………….

19. Please list any previous medication/s you have taken specifically for your arthritis in the last 10 years.

Medication examples include: Prednisolone, diclofenac, naproxen, methotrexate, sulfasalazine (Azulfidine®), hydroxychloroquine (Plaquenil®), leflunomide (Arava®), abatacept (Orencia®), rituximab (Rituxan®), tocilizumab (Actemra®), sarilumab (Kevzara®), anakinra (Kineret®), adalimumab (Humira®), etanercept (Enbrel®), infliximab (Remicade®), certolizumab pegol (Cimzia®), golimumab (Simponi®), ixekizumab (Taltz®), secukinumab (Cosentyx®), ustekinumab (Stelara®), guselkumab (Tremfya®), gold injections (Myocrisin®), auranofin (Ridaura®), azathioprine (Imuran®, Azasan®), cyclosporine (Gengraf®, Neoral®, Sandimmune®) and apremilast (Otezla®)

………………………………………………………………………………………….

………………………………………………………………………………………….

………………………………………………………………………………………….

**Awareness of JAK inhibitors**

20. How aware are you of JAK inhibitors?

Very aware

Somewhat aware

Not aware

Not very aware

Not aware at all

21. From which source/s did you hear about JAK inhibitors (if applicable)?

Rheumatologist

Rheumatology Specialist Nurse

Patient organisation

Family/friends

Newspapers

Internet

Social media

Other – please state: ………………………………………………………………...

22. If on a biologic, would you prefer an oral therapy instead (such as a JAK inhibitor) to control your arthritis?

Yes

No

Don’t know

Not applicable as already tried a JAK inhibitor

1. Is there anything else (not mentioned elsewhere) that you would like to add?

…………………………………………………………………………………….… ………………………………………………………………………………………. ………………………………………………………………………………………. ……………………………………………………………………………………….

**Further contact (Optional)**

Please leave your email address if you wish to be contacted for any of the purposes below (select all that apply):


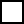
 To receive a summary of the study results (expected Spring 2023)


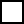
 To enter a prize draw to win one of 3 x £100 Love2Shop digital gift cards


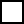
 To participate in a 1.5 hours online patient focus group on your views and experiences of JAK inhibitors (payment of a £40 Love2Shop digital gift card after focus group) - Only patients who have current or previous use of JAK inhibitors are eligible for this part of the study

Email address: ………………………………………………………………………………..

Thank you for your participation!

**Please click ‘submit’ to complete survey.**
