# Supplementary material for: Clinician and patient views on janus kinase inhibitors in the treatment of inflammatory arthritis: a mixed methods study
Source: BMC Rheumatol. 2024 Jan 17;8:1. doi: 10.1186/s41927-023-00370-7 (PMC10792861; doi:10.1186/s41927-023-00370-7)
Supplement: Supplementary file 3 — Additional file 3. Patient interview and focus group topic guide [file 41927_2023_370_MOESM3_ESM.docx]

**Patient interview and focus group topic guide**

1) Why was a JAK inhibitor prescribed for you?

2) What expectations did you have before starting a JAK inhibitor?

3) Has starting a JAK inhibitor affected your lifestyle in any way? (Pros/cons)

4) Have you encountered any difficulties since using JAK inhibitors or had to stop taking them?

5) Do you have any concerns over the use of JAK inhibitors? (Short-term/long-term, safety)

6) How do JAK inhibitors compare to previous treatments for your inflammatory arthritis?

7) Has the COVID-19 pandemic made you think differently about JAK inhibitors?

8) Anything else you would like to mention?
