# Supplementary material for: Clinician and patient views on janus kinase inhibitors in the treatment of inflammatory arthritis: a mixed methods study
Source: BMC Rheumatol. 2024 Jan 17;8:1. doi: 10.1186/s41927-023-00370-7 (PMC10792861; doi:10.1186/s41927-023-00370-7)
Supplement: Supplementary file 4 — Additional file 4. Clinician survey demographics [file 41927_2023_370_MOESM4_ESM.docx]

| **Clinician characteristics** | **n (%)**  **N= 51** |
| --- | --- |
| *Role*  Rheumatology Consultant  Rheumatology Registrar  Rheumatology Clinical Nurse  Clinical Fellow  Other rheumatology role | 37 (72.6)  7 (13.7)  5 (9.8)  1 (2.0)  1 (2.0) |
| *UK region of practice*  North East England  North West England  Yorkshire and the Humber  West Midlands  East Midlands  South West England  South East England  East of England  Greater London  Wales  Scotland  Northern Ireland | 3 (5.9)  8 (15.7)  6 (11.8)  3 (5.9)  1 (2.0)  2 (3.9)  4 (7.8)  7 (13.7)  9 (17.7)  0 (0.0)  6 (11.8)  2 (3.9) |
| *Work setting*  Secondary care  Tertiary care  Other | 35 (68.6)  16 (31.4)  0 (0.0) |
| *Time spent on research as part of job*  0%  1-25%  26-50%  >50% | 14 (27.5)  20 (39.2)  7 (13.7)  10 (19.6) |
| *%age of RA/PsA patients on a JAKi*  1%  1-5%  6-10%  11-15%  16-20%  >20% | 3 (5.9)  26 (51.0)  12 (23.5)  10 (19.6)  0 (0.0)  0 (0.0) |

**Clinician survey demographics**

JAKi = janus kinase inhibitor; PsA = psoriatic arthritis; RA = rheumatoid arthritis
