# Supplementary material for: Clinician and patient views on janus kinase inhibitors in the treatment of inflammatory arthritis: a mixed methods study
Source: BMC Rheumatol. 2024 Jan 17;8:1. doi: 10.1186/s41927-023-00370-7 (PMC10792861; doi:10.1186/s41927-023-00370-7)
Supplement: Supplementary file 5 — Additional file 5. Clinician survey additional comments [file 41927_2023_370_MOESM5_ESM.docx]

| **Clinician survey - Additional comments** |
| --- |
| “The thrombotic risk may well be a class effect so we are counselling patients.” - Clinician 1 (Consultant, East of England)  “I’ve had a bad run, 2/5 patients on tofacitinib have had VTE. That said for two others they [JAKi] have been an absolute god send.” - Clinician 4 (Consultant, East of England)  “I think JAKi are effective drugs, but despite the drug reps [representatives] trying to reassure us and convince to prescribe there is still a big safety concern, particularly when there are so many other therapies available. I will not be prescribing at large scale until more data is available.” - Clinician 9 (Consultant, East of England)  “Concerns about cardiovascular safety” - Clinician 10 (Clinical Fellow, Scotland)  “Recent safety data (ORAL surveillance) and subsequent MHRA advice is likely to significantly reduce prescribing of JAKi.” - Clinician 12 (Consultant, North West England)  “Recent safety concerns from ORAL Surveillance makes prescribing [JAKi] harder. That population had CV [cardiovascular] risk factors which makes generalizability difficult.” - Clinician 18 (Consultant, North West England)  “I often prescribe JAKi in patients with needle phobia. My general feeling is that they tend to lose efficacy after a few years. Recent concerns about clots has limited who we can start these medicines in.” - Clinician 21 (Registrar, Greater London)  “Anecdotally a few patients have developed cancer on JAKi - have yellow carded this.” - Clinician 24 (Registrar, Greater London)  “The latest MHRA alert will reduce my prescribing of JAKs.” - Clinician 25 (Consultant, Yorkshire)  “Worrying data re [regarding] malignancy with Tofacitinib.” - Clinician 26 (Consultant, Greater London)  “Provide an effective therapeutic option in patients with disease resistant to other therapies. Long term safety data is a concern but awaiting upa and filgotinib data with interest.” - Clinician 31 (Registrar, Greater London)  “I am concerned about recent reports of increased VTE and malignancies.” - Clinician 34 (Consultant, Yorkshire)  “Historically skin efficacy for PsA for tofa has meant used less and upa only recently available which impacted on JAKi use in PsA.” - Clinician 35 (Consultant, Scotland)  “Concerned about new black box warnings, CVD [cardiovascular disease], etc.I was expecting JAKs to take off this year, but with the increased risk of covid as well it’s a worry.” - Clinician 46 (Consultant, Northern Ireland) |

covid = coronavirus disease; JAKi = janus kinase inhibitor; MHRA = Medical Health and Regulatory Authority (UK); PsA = psoriatic arthritis; tofa = tofacitinib; upa = upadacitinib; VTE = venous thromboembolism
