# Supplementary material for: Clinician and patient views on janus kinase inhibitors in the treatment of inflammatory arthritis: a mixed methods study
Source: BMC Rheumatol. 2024 Jan 17;8:1. doi: 10.1186/s41927-023-00370-7 (PMC10792861; doi:10.1186/s41927-023-00370-7)
Supplement: Supplementary file 6 — Additional file 6. Patient survey demographics [file 41927_2023_370_MOESM6_ESM.docx]

**Patient survey demographics**

| **Patient characteristics** | **Mean, s.d or n (%)**  **N=141** |
| --- | --- |
| *Age (years)* | 52.6, 12.4 |
| *Gender*  Male  Female  Non-binary  Other | 11 (7.8)  130 (92.2)  0 (0.0)  0 (0.0) |
| *Ethnicity*  White  Black/Minority | 133 (94.3)  8 (5.7) |
| *UK region*  North East England  North West England  Yorkshire and the Humber  West Midlands  East Midlands  South West England  South East England  East of England  Greater London  Wales  Scotland  Northern Ireland | 6 (5.7)  12 (8.5)  7 (5.0)  12 (8.5)  12 (8.5)  17 (12.1)  19 (13.5)  11 (7.8)  15 (10.6)  9 (6.4)  13 (9.2)  6 (4.3) |
| *Employment status*  Employed full-time  Employed part-time  Self-employed  Unemployed  Retired  Student  Other | 36 (25.5)  28 (19.9)  12 (8.5)  12 (8.5)  40 (28.4)  2 (1.4)  11 (7.8) |
| *Job type*  Manual  Non-Manual  Other  Not applicable – do not work | 4 (2.8)  79 (56.0)  1 (0.7)  57 (40.4) |
| *Clinical diagnosis*  Rheumatoid arthritis  Psoriatic arthritis | 104 (73.8)  37 (26.2) |
| *Disease duration (years)* | 14, 10.5 |
| *Medications**  JAKi  Tofacitinib  Baricitinib  Upadacitinib  Filgotinib  Biologic by self-injection  Biologic by infusion  Methotrexate in tablet form  Methotrexate as an injection  Sulfasalazine  Hydroxychloroquine  Leflunomide  Other | 56 (39.7)  13 (23.2)  34 (60.7)  4 (7.1)  5 (8.9)  68 (48.2)  16 (11.3)  17 (12.1)  36 (25.5)  22 (15.6)  16 (11.3)  12 (8.5)  12 (8.5) |

*can be on more than one; JAKi = janus kinase inhibitor
