# Supplementary material for: Clinician and patient views on janus kinase inhibitors in the treatment of inflammatory arthritis: a mixed methods study
Source: BMC Rheumatol. 2024 Jan 17;8:1. doi: 10.1186/s41927-023-00370-7 (PMC10792861; doi:10.1186/s41927-023-00370-7)
Supplement: Supplementary file 7 — Additional file 7. Patient survey - Why did you stop taking the JAK inhibitor? [file 41927_2023_370_MOESM7_ESM.docx]

**Patient survey - Why did you stop taking the JAK inhibitor?**

| **Themes** | **All responses** |
| --- | --- |
| Inefficacy | “It did not control my RA…” - Patient 4 (Baricitinib, RA)  “Stopped working.” - Patient 38 (Tofacitinib, PsA)  “...it wasn't as effective as Toczilizumab has previously been for me.”  - Patient 46 (Tofacitinib, RA)  “Active arthritis.” - Patient 60 (Tofacitinib, PsA)  “Didn’t work.” - Patient 79 (Tofacitinib, RA)  “Not effective.” - Patient 81 (Tofacitinib, RA)  “Ceased effectiveness.” - Patient 83 (Baricitinib, RA)  “It did not work.” - Patient 116 (Tofacitinib, PsA)  “Ineffective.” - Patient 127 (Baricitinib, RA)  “It wasn’t effective.” - Patient 130 (Tofacitinib, PsA) |
| Non-MACE adverse effects | “...and a major side effect was that it made me feel generally unwell.”  - Patient 4 (Baricitinib, RA)  “Side effects were diarrhoea for a prolonged amount of time (weeks), vomiting, stomach pain and discomfort.” - Patient 41 (Filgotinib, RA)  “Inflammatory markers were high, wasn't feeling the best...” - Patient 46 (Tofacitinib, RA)  “The XXX [hospital] clinic asked me to stop after having had blood test.”  - Patient 87 (Unknown JAKi, RA)  “Side effect..leg muscles sore felt like severe bruising and total fatigue of lower leg, felt unable to walk..GP advised me to stop meds.” - Patient 93 (Tofacitinib, PsA)  “It made me neutropenic.” - Patient 123 (Unknown JAKi, RA)  “Neutropenia.” - Patient 133 (Tofacitinib, PsA)  “It gave me continuous chest infections and pneumonia.” - Patient 139 (Baricitinib, RA) |
| MACE | “Advised to stop in hospital for heart attack.” - Patient 86 (Tofacitinib, RA) |

GP = General Practitioner; JAKi = janus kinase inhibitor; MACE = major adverse cardiovascular

event; PsA = psoriatic arthritis; RA = rheumatoid arthritis
