# Supplementary material for: Clinician and patient views on janus kinase inhibitors in the treatment of inflammatory arthritis: a mixed methods study
Source: BMC Rheumatol. 2024 Jan 17;8:1. doi: 10.1186/s41927-023-00370-7 (PMC10792861; doi:10.1186/s41927-023-00370-7)
Supplement: Supplementary file 8 — Additional file 8. Patient interview and focus group demographics [file 41927_2023_370_MOESM8_ESM.docx]

| **Patient characteristics** | **Mean, s.d. or n (%)**  **N = 18** |
| --- | --- |
| *Age (years)* | 57.6, 11.6 |
| *Gender*  Male  Female | 3 (16.7)  15 (83.3) |
| *Ethnicity*  White  Black/Minority | 18 (100.0)  0 (0.0) |
| *UK region*  North East England  North West England  Yorkshire and the Humber  West Midlands  East Midlands  South West England  South East England  East of England  Greater London  Wales  Scotland  Northern Ireland | 1 (5.6)  4 (22.2)  0 (0.0)  0 (0.0)  0 (0.0)  3 (16.7)  2 (11.1)  1 (5.6)  1 (5.6)  1 (5.6)  2 (11.1)  3 (16.7) |
| *Employment status*  Employed full-time  Employed part-time  Self-employed  Not in employment  Unable to work  Retired  Voluntary work | 2 (11.1)  2 (11.1)  1 (5.6)  1 (5.6)  2 (11.1)  8 (44.4)  2 (11.1) |
| *Clinical diagnosis*  Rheumatoid arthritis  Psoriatic arthritis  Rheumatoid and psoriatic arthritis | 14 (77.8)  3 (16.7)  1 (5.6) |
| *Disease duration (years)* | 17, 10.0 |
| *Current JAKi*  Tofacitinib  Baricitinib  Upadacitinib  Filgotinib | 7 (38.9)  7 (38.9)  1 (5.6)  2 (11.1) |
| *Time on current JAKi (years)* | 3, 1.1 |
| *Current biologic* | 1 (5.6) |
| *Concurrent cDMARDs/steroids* | 11 (61.1) |
| *Last medication prior to current therapy*  JAKi (baricitinib)  Biologic  cDMARDs and steroids  cDMARDs only | 1 (5.6)  15 (83.3)  1 (5.6)  1 (5.6) |

**Patient interview and focus group demographics**

cDMARDS = conventional disease-modifying antirheumatic

drugs; JAKi = janus kinase inhibitor
