# Supplementary material for: Clinician and patient views on janus kinase inhibitors in the treatment of inflammatory arthritis: a mixed methods study
Source: BMC Rheumatol. 2024 Jan 17;8:1. doi: 10.1186/s41927-023-00370-7 (PMC10792861; doi:10.1186/s41927-023-00370-7)
Supplement: Supplementary file 9 — Additional file 9. Theme 1: Journey onto JAKi [file 41927_2023_370_MOESM9_ESM.docx]

**Theme 1: Journey onto JAKi**

| **Subthemes** | **Illustrative quotes** |
| --- | --- |
| Decision-making process | “...I had a long conversation with the rheumatology specialist nurse. It was a phone call conversation about why the consultant was prescribing that particular one [tofacitinib]…” - Patient 11 (RA & PsA, South West England)  “I think that at the time that I was prescribed it [baricitinib], I probably knew more about it than the consultant did.” - Patient 13 (RA, Scotland)  “I didn’t get any…information at all from the hospital….” - Patient 9 (RA, North West England)  “All my joints were very swollen and my consultant said, ‘We will put you on baricitinib…’. There was no discussion.” - Patient 16 (RA, South West England)  “I kind of feel that they are the consultant, and they know more, but they give us the choice [of medications] and I don’t feel that I know enough…That is how I feel, in that they [clinicians] should choose.” - Patient 5 (RA, Northern Ireland) |
| Why JAKi prescribed | “So, it was a case of having tried a lot of the other drugs and she [rheumatologist] thought that this [baricitinib] was worth a go.”- Patient 3 (RA, North West England)  “The reason why we changed from that [rituximab]…was that my psoriatic side started to show a lot of symptoms. I was getting rashes all over my hands and my nails were all falling off…my consultant…said she would think about the one drug that would target both my rheumatoid and my psoriatic [arthritis]...” - Patient 11 (RA & PsA, South West England)  “...my rheumatologist would be canny enough to say to me that if they can get me stabilised on this drug treatment [tofacitinib], it would be more cost-effective, because anything that is going to involve me going to have infusions or involving another member of staff in the health service giving me an injection,…all the room space and the personnel that is used…and ultimately the drugs [JAKi] would come off patent and it would be cheaper in the end.” - Patient 4 (RA, Northern Ireland) |
| Expectations of the JAKi | “I think that my expectations were quite low because every drug that I had been on hadn’t really been of a great help. So, I was just hoping that the pain I had been living with was going to stop and thankfully it did.” - P12 (RA, North West England)  “My expectation was that it [JAKi] would get my disease back under control again…” - Patient 16 (RA, South West England)  “Each time I was put onto an injectable biologic, they started to work fairly quickly and brought me into remission for a long time and I was hopeful that the same thing would happen with the JAK inhibitor, that it would bring me into remission.” - Patient 15 (RA, East of England)  “...I got a bit concerned about the side effects [of the JAK] from what I read.” - Patient 9 (RA, North West England)  “...with having had shingles [herpes zoster] previously [on rituximab], my concern was would I end up with shingles again [on tofacitinib], because…that [shingles] is one of the things in the information [leaflet]…” - Patient 11 (RA & PsA, South West England) |
| Awareness of and access to JAKi | “...non-existent [GP knowledge of JAKi]. Even pharmacists are well like, ‘what the hell are you on?’.” - Patient 1 (PsA, Greater London)  “ I had breast cancer last Summer,…but the oncologist knew about JAKs, interestingly.” - Patient 2 (RA, South East England)  “...too many people do not know about JAKs…There seems to be a huge reluctance by rheumatologists generally to prescribe them…there is a lot of damage to joints being done, whereby it could be helped. I mean, in the long-term that costs the NHS, so much more money than it [JAKi] being available…I know that there are the NICE [National Institute for Health and Care Excellence] guidelines and…that you have to with the old biologics…fail a certain amount in order to get on it [JAKi], but from a patient perspective I just think that they [JAKi] should be much more readily available and consultants should be a bit more willing to consider them.” - Patient 1 (PsA, Greater London) |

JAKi = janus kinase inhibitor; PsA = psoriatic arthritis; RA = rheumatoid arthritis
