# Supplementary material for: Clinician and patient views on janus kinase inhibitors in the treatment of inflammatory arthritis: a mixed methods study
Source: BMC Rheumatol. 2024 Jan 17;8:1. doi: 10.1186/s41927-023-00370-7 (PMC10792861; doi:10.1186/s41927-023-00370-7)
Supplement: Supplementary file 10 — Additional file 10. Theme 2: Experience of using JAKi [file 41927_2023_370_MOESM10_ESM.docx]

**Theme 2: Experience of using JAKi**

| **Subthemes** | **Illustrative quotes** |
| --- | --- |
| Time to take effect | “...as soon as I went on it [baricitinib], it made a big difference straight away; within days, I felt so much better.” - Patient 9 (RA, North West England)  “ I felt fantastic within three or four days of taking it [filgotinib], and unlike the change over with the other ones [previous biologics], the nurse said that it would work quite quickly. So, they didn’t give me a short course of prednisolone tablets, which they did with the others [previous biologics]….”  - Patient 15 (RA, East of England)  “So, it [tofacitinib] took…a long time to kind of kick in. I could feel that it [PsA] was getting better, but it did take an awfully long time for it [tofacitinib] to work…Maybe six to eight months.” - Patient 1 (PsA, Greater London) |
| Quality of life | “ But the one that I have got now [filgotinib]…it just has had a massive impact on my life. It has given me that chance to go back to being someone who I am and who I want to be, not someone who is ruled by their disease and their condition…This time last year…I could not…put my shoes on, and I couldn’t even dress myself…and now literally, I am climbing mountains…I am at the gym…three or four times a week and I am with a personal trainer for another couple of times a week…I am actually able to get on with things and do things that I enjoy and actually have a life.” - Patient 7 (RA, Wales)  “I think the pros would be the change, the total turnaround in my mobility and able to enjoy life. So much better and able to get out and about, and able to get out and walk every day. That is the big difference that it [baricitinib] has made for me.” - Patient 8 (RA, Northern Ireland)  “ I have had difficulty keeping my weight down, and I think that is a very common finding amongst other people [on JAKi].” Patient 13 (RA, Scotland)  “...it [tofacitinib] has certainly improved my quality of life…I feel that this might be a keeper for positive planning. For all of us, one of the things that RA took away was being able to plan confidently…That is really something when you can start believing that can happen again. That is really great.”  - Patient 4 (RA, Northern Ireland)  “…certainly at the moment, it [baricitinib] has secured my employment, and I have been able to stay in work, and increasing my hours.” - Patient 10 (RA, Northern Ireland) |
| Comparison with previous treatments | “ It [tofacitinib] is much easier, as I was getting needle fear from some of the other ones…and I would get to the point where I could not bear to inject anything. So, to have a pill was marvellous.” - Patient 1 (PsA, Greater London)  “ The pro is also the ease that you are taking it [baricitinib] by tablet form and you are not going into the hospital and taking a half day to have your infusions.” - Patient 10 (RA, Northern Ireland)  “I would say that they [JAKi] are very easy to take, having been on self-injectables and I have gone in for infusions, and taking a tablet [JAKi] twice a day is very easy alongside my other medications.” - Patient 11 (RA & PsA, South West England)  “...going to visit my mum…it meant that if it was an injection day when I was with her, I did not have to think of taking the injection with me and putting it in the fridge and having the sharps container or anything like that. So, it [filgotinib] was very convenient.” - Patient 15 (RA, East of England)  “ It [baricitinib] is great when you are travelling, rather than having to go through all the letters and being asked at Customs, ‘What is this, and what is this injection doing here?’. So it is much more convenient to take.” - Patient 3 (RA, North West England) |
| Side effects | “...so pros [of taking tofacitinib] very few side effects. So, absolutely, very few side effects, and that is a real win for me.” - Patient 1 (PsA, Greater London)  “ …I haven’t had any negative reactions at all really. So, it [baricitinib] has been a very positive experience for me.” - Patient 3 (RA, North West England)  “My cholesterol level is quite high now [since taking baricitinib] to the extent that I am now taking statins, which I never thought that I would have to, to reduce the overall cholesterol level.” - Patient 13 (RA, Scotland)  “Since being on it [tofacitinib], I would say that I have the most uncontrollable hair a person could ever have. I actually wake up and it is a mass of frizz…I do now suffer, like I never did before, with a very dry mouth and I have suffered with urine infections, and repeated ones.” - Patient 12 (RA, North West England)  “My cons [with being on tofacitinib] are that I do have brain fog and memory issues. I can forget what I am going to do tomorrow…” - Patient 5 (RA, Northern Ireland) |
| Stopping and switching JAKi | “...I was only on it [baricitinib] for about three weeks, and I had the mother of all cold sores..and it was horrendous, and I had to come off it and I was given an antiviral for that. And I have had one or two cold sores since that now… I also have had to come off it, because of a urinary tract infection, but that happened to me when I was on other drugs as well. But I have never had the cold sore issue before. So, that was definitely something that was related to the current drug [baricitinib].” - Patient 8 (RA, Northern Ireland)  “Occasionally, I get the odd chest infection which means [taking] antibiotics…you have to stop taking your JAKs…so that your…antibiotics can work.” - Patient 6 (RA, South West England)  “ I went onto baricitinib and that immediately gave me severe neutropenia…then I got to a point when I started to feel really unwell and got quite a few infections, and then they changed me over to tocilizumab [a biologic], which is what I am taking and injecting with myself now. As well as my white [cell] blood count, I had problems with my liver enzymes…they were raised, and that was another reason why I had to stop taking it [baricitinib]. ” - Patient 16 (RA, South West England)  “ I commenced the treatment [tofacitinib] in August and until the following April or May…that was when I started to have the [heart] palpitations and the atrial fibrillation… They [rheumatology team] shifted me over to baricitinib…and so far, I am tolerating that well with no side effects…” - Patient 4 (RA, Northern Ireland) |

JAKi = janus kinase inhibitor; PsA = psoriatic arthritis; RA = rheumatoid arthritis
