# Supplementary material for: Clinician and patient views on janus kinase inhibitors in the treatment of inflammatory arthritis: a mixed methods study
Source: BMC Rheumatol. 2024 Jan 17;8:1. doi: 10.1186/s41927-023-00370-7 (PMC10792861; doi:10.1186/s41927-023-00370-7)
Supplement: Supplementary file 11 — Additional file 11. Theme 3: Concerns over using JAKi [file 41927_2023_370_MOESM11_ESM.docx]

**Theme 3: Concerns over using JAKi**

| **Subthemes** | **Illustrative quotes** |
| --- | --- |
| Safety concerns of patients | “...when I was warned that my cholesterol was likely to rise on the filgotinib,…that certainly worried me because all four of my grandparents died from cardiovascular disease.” - Patient 15 (RA, East of England)  “...and the possibility of cancer, whether it [tofacitinib] raises the risk with cancer.” - Patient 12 (RA, North West England)  “... there are not enough longitudinal studies [on JAKi], but anything that I have been reading about risk, I am still seeing very small, very, very small percentages of concern.” - Patient 4 (RA, Northern Ireland) |
| Safety concerns of health professionals | “My pharmacist from the GP [general practice surgery], she did ring me up with a concern that I was taking it [baricitinib], and had I looked at all the side effects? And when I went to see the orthopaedic surgeon about me having the hip replacement, and he was shaking his head and saying, ‘that is serious stuff that you are taking there [baricitinib] with the methotrexate…’.” - Patient 9 (RA, North West England)  “...when I think that a consultant phones you up and says that he is concerned about some recent studies and wants to take you off the drug, I think that it is a cause for concern. Even though it is not baricitinib in the study [ORAL Surveillance], it is the group [JAKi].” - Patient 14 (RA, North West England)  “ …the whole of the rheumatologists in Northern Ireland withdrew tofacitinib and changed the patients onto baricitinib.” - Patient 4 (RA, Northern Ireland) |
| Patient attitudes towards JAKi use | “I believe that these drugs [JAKi] are safe. I just believe there are risks associated with them, as there are with any medication.” - Patient 18 (PsA, North East England)  “ I suppose at the back of your mind there is always a slight concern that it [baricitinib]…has not been used for that a long time. So, no one really knows the long-term effect of it do they? But the advantages outweigh those concerns for me.” - Patient 3 (RA, North West England)  “I would rather have the benefit, than the risk. Yes, I appreciate that there might be long-term issues, but I think that anyone with a long-term chronic disease understands that they have to live today rather than tomorrow. So, if it [tofacitinib] takes me out tomorrow, then at least I had today, and it wasn’t too bad. So, I will take that.” - P1 (PsA, Greater London)  “I mean it [tofacitinib] is great…I have been on it for three years, and I have started to notice in recent months that I have started to get more flare ups again and more regularly…mild ones, but I get the aches and pains…So, I do wonder whether it is going to be effective in another two or three years’ time.” - Patient 6 (RA, South West England) |

JAKi = janus kinase inhibitor; PsA = psoriatic arthritis; RA = rheumatoid arthritis
