# Supplementary material for: Clinician and patient views on janus kinase inhibitors in the treatment of inflammatory arthritis: a mixed methods study
Source: BMC Rheumatol. 2024 Jan 17;8:1. doi: 10.1186/s41927-023-00370-7 (PMC10792861; doi:10.1186/s41927-023-00370-7)
Supplement: Supplementary file 12 — Additional file 12. Theme 4: JAKi and the COVID-19 pandemic [file 41927_2023_370_MOESM12_ESM.docx]

**Theme 4: JAKi and the COVID-19 pandemic**

| **Subthemes** | **Illustrative quotes** |
| --- | --- |
| Attitudes towards JAKi during the pandemic | “...I actually stopped [filgotinib] for a little while…All you can think about is is my drug making me more susceptible?…I was so fearful that anybody that I came into contact who had it [COVID-19], it would put me in intensive care basically…Now I would not stop taking it [filgotinib]…but initially [height of the COVID-19 pandemic] it would get to the point where I was having a panic attack with just taking the medication…I could not think clearly, because I think we were being told so much that you are immunocompromised, and you are on a medication that makes you more susceptible.” - Patient 7 (RA, Wales)  “...the fact that JAK inhibitors, like any immunosuppressive, make you much more vulnerable to COVID-19 is a worry, but again, having better symptom control was a far greater priority for me than worrying about whether I had to shield or not.” - Patient 18 (PsA, North East England)  “I had my fourth booster [COVID-19 vaccination] and from everything that I have read, I did not take my methotrexate for a week and I did not take my baricitinib for a week…” - Patient 4 (RA, Northern Ireland)  “ I had caught…COVID very early on in the pandemic…so I did come off the baricitinib for about ten days, but not the methotrexate.”- Patient 2 (RA, South East England) |
| Starting a JAKi during the pandemic | “ I think that I was shifted onto the JAK [tofacitinib], because my flare up came at the time of the lockdown and they [clinicians] wanted to choose something that would be a tablet, so that I did not have to travel anywhere and expose myself to the risk of infection.” - Patient 4 (RA, Northern Ireland)  “NRAS [National Rheumatoid Arthritis Society] had…mentioned that rituximab could be a problem with the [COVID-19] vaccines and so the hospital said eventually that I could have baricitinib instead of rituximab, because…it didn’t knock out all your B cells for six months, with what they were saying with the rituximab.” Patient 9 (RA, North West England) |
| Potential benefits of JAKi during the pandemic | “...you are only taking a tablet or maybe two tablets a day, you can come off that [JAKi] very quickly…So, that gave me good heart actually, that if I did catch COVID I am not on something like when I was on the biologics…I took the injection once a week. So, I had that in my system for that length of time. If I had an infusion, it would be in my system for so much longer, whereas this [JAKi] has a shorter term in your system. So, if you have to come off it, you are in a better position…than what you would have been if you had been on the biologic.” - Patient 8 (RA, Northern Ireland)  “I heard that it [COVID-19] was a systemic and inflammatory disease with a viral trigger…and I was thinking…I might actually be protected by baricitinib... ” - P13 (RA, Scotland) |

COVID-19 = coronavirus disease 2019; JAKi = janus kinase inhibitor; PsA = psoriatic arthritis; RA = rheumatoid arthritis
